# Supplementary material for: Mitochondrial genome of the nonphotosynthetic mycoheterotrophic plant Hypopitys monotropa, its structure, gene expression and RNA editing
Source: PeerJ. 2020 Jun 19;8:e9309. doi: 10.7717/peerj.9309 (PMC7307570; doi:10.7717/peerj.9309)

Supplementary figure 5.

Phylogenetic trees inferred from ML analysis of single mitochondrial genes. Protein coding genes that were annotated in mitochondrial genomes of *Hypopitys monotropa, Vaccinium macrocarpon* and all main evolutionary lineages of flowering plants are included in the analysis.

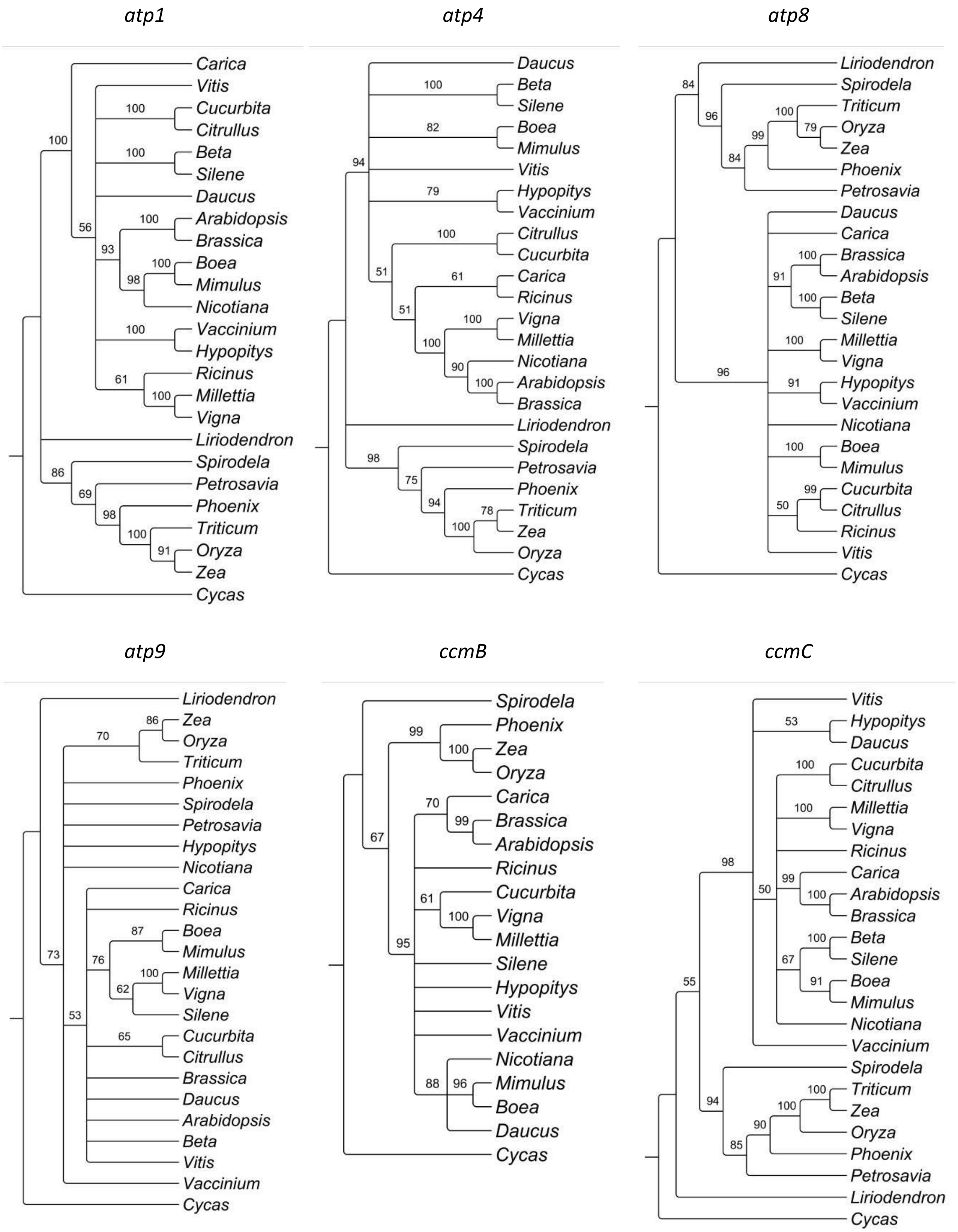

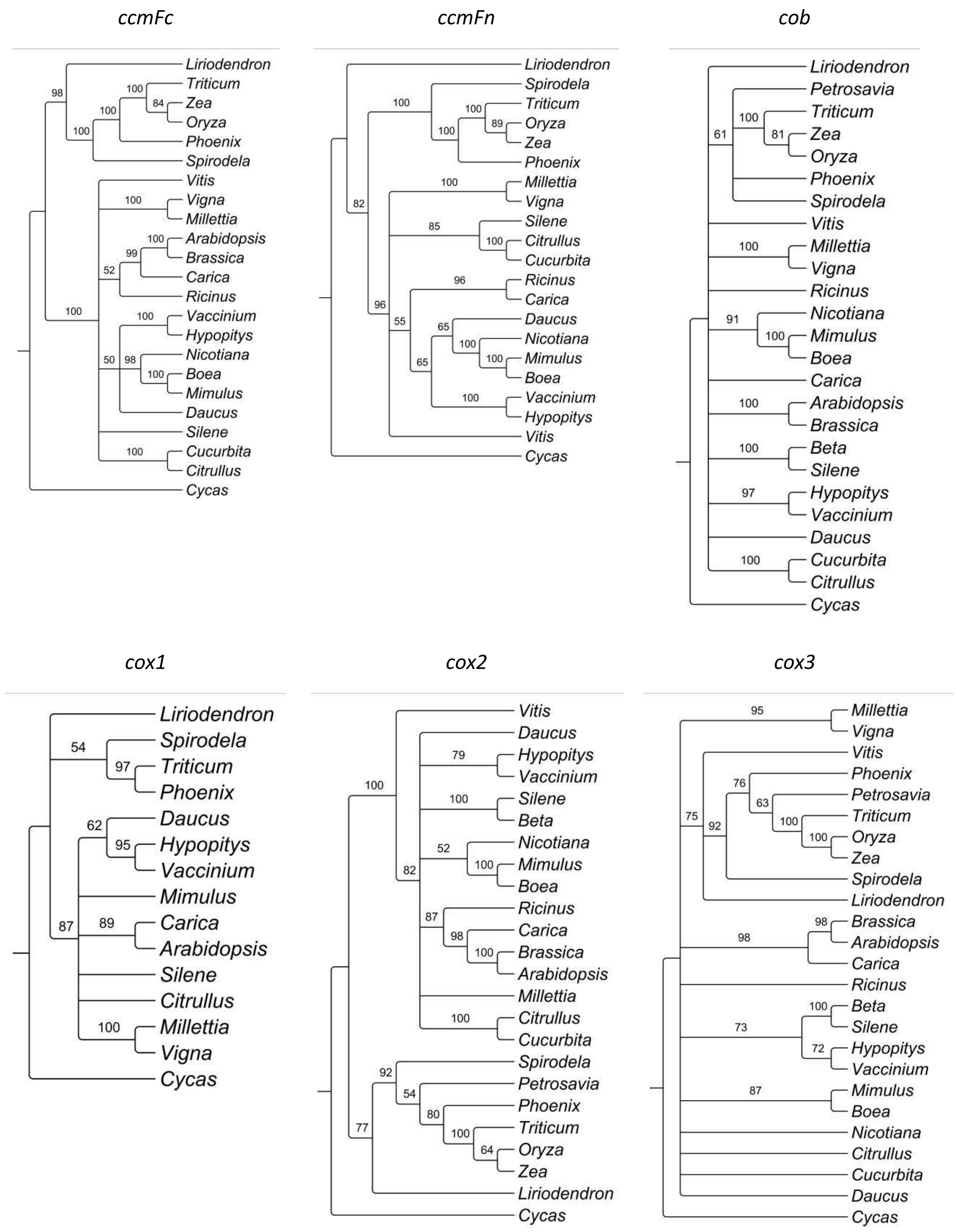

ccmFc
Liriodendron
Triticum
Zea
Oryza
Phoenix
Spirodela
Vitis
Vigna
Millettia
Arabidopsis
Brassica
Carica
Ricinus
Vaccinium
Hypopitys
Nicotiana
Boea
Mimulus
Daucus
Silene
Cucurbita
Citrullus
Cycas
ccmFn
Liriodendron
Spirodela
Triticum
Oryza
Zea
Phoenix
Millettia
Vigna
Silene
Citrullus
Cucurbita
Ricinus
Carica
Daucus
Nicotiana
Mimulus
Boea
Vaccinium
Hypopitys
Vitis
Cycas
cob
Liriodendron
Petrosavia
Triticum
Zea
Oryza
Phoenix
Spirodela
Vitis
Millettia
Vigna
Ricinus
Nicotiana
Mimulus
Boea
Carica
Arabidopsis
Brassica
Beta
Silene
Hypopitys
Vaccinium
Daucus
Cucurbita
Citrullus
Cycas
cox1
Liriodendron
Spirodela
Triticum
Phoenix
Daucus
Hypopitys
Vaccinium
Mimulus
Carica
Arabidopsis
Silene
Citrullus
Millettia
Vigna
Cycas
cox2
Vitis
Daucus
Hypopitys
Vaccinium
Silene
Beta
Nicotiana
Mimulus
Boea
Ricinus
Carica
Brassica
Arabidopsis
Millettia
Citrullus
Cucurbita
Spirodela
Petrosavia
Phoenix
Triticum
Oryza
Zea
Liriodendron
Cycas
cox3
Millettia
Vigna
Vitis
Phoenix
Petrosavia
Triticum
Oryza
Zea
Spirodela
Liriodendron
Brassica
Arabidopsis
Carica
Ricinus
Beta
Silene
Hypopitys
Vaccinium
Mimulus
Boea
Nicotiana
Citrullus
Cucurbita
Daucus
Cycas

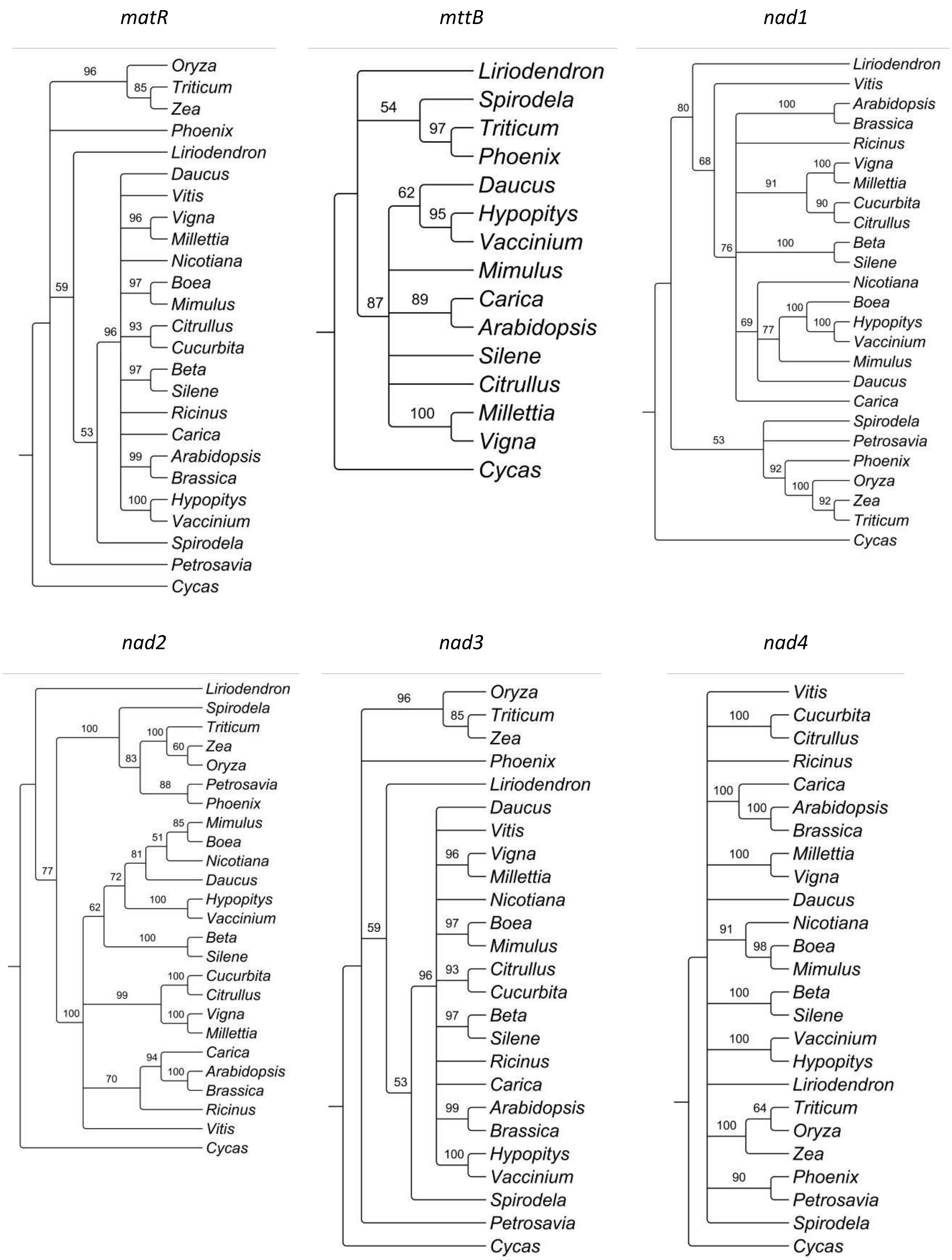
matR
96
Oryza
85
Triticum
Zea
Phoenix
Liriodendron
Daucus
Vitis
96
Vigna
Millettia
Nicotiana
97
Boea
Mimulus
59
93
Citrullus
96
Cucurbita
97
Beta
Silene
Ricinus
53
Carica
99
Arabidopsis
Brassica
100
Hypopitys
Vaccinium
Spirodela
Petrosavia
Cycas
mttB
Liriodendron
54
Spirodela
97
Triticum
Phoenix
62
Daucus
95
Hypopitys
Vaccinium
Mimulus
87
89
Carica
Arabidopsis
Silene
Citrullus
100
Millettia
Vigna
Cycas
nad1
Liriodendron
Vitis
80
100
Arabidopsis
Brassica
Ricinus
68
100
Vigna
91
Millettia
90
Cucurbita
Citrullus
76
100
Beta
Silene
Nicotiana
100
Boea
69
77
100
Hypopitys
Vaccinium
Mimulus
Daucus
Carica
Spirodela
53
Petrosavia
92
Phoenix
100
Oryza
92
Zea
Triticum
Cycas
nad2
Liriodendron
Spirodela
100
100
Triticum
60
Zea
83
Oryza
88
Petrosavia
Phoenix
85
Mimulus
51
Boea
81
Nicotiana
72
Daucus
77
100
Hypopitys
62
Vaccinium
100
Beta
Silene
100
Cucurbita
99
Citrullus
100
100
Vigna
Millettia
94
Carica
100
Arabidopsis
70
Brassica
Ricinus
Vitis
Cycas
nad3
96
Oryza
85
Triticum
Zea
Phoenix
Liriodendron
Daucus
Vitis
96
Vigna
Millettia
Nicotiana
59
97
Boea
Mimulus
96
93
Citrullus
Cucurbita
97
Beta
Silene
Ricinus
53
Carica
99
Arabidopsis
Brassica
100
Hypopitys
Vaccinium
Spirodela
Petrosavia
Cycas
nad4
Vitis
100
Cucurbita
Citrullus
Ricinus
100
Carica
100
Arabidopsis
Brassica
100
Millettia
Vigna
Daucus
91
Nicotiana
98
Boea
Mimulus
100
Beta
Silene
100
Vaccinium
Hypopitys
Liriodendron
64
Triticum
100
Oryza
Zea
90
Phoenix
Petrosavia
Spirodela
Cycas

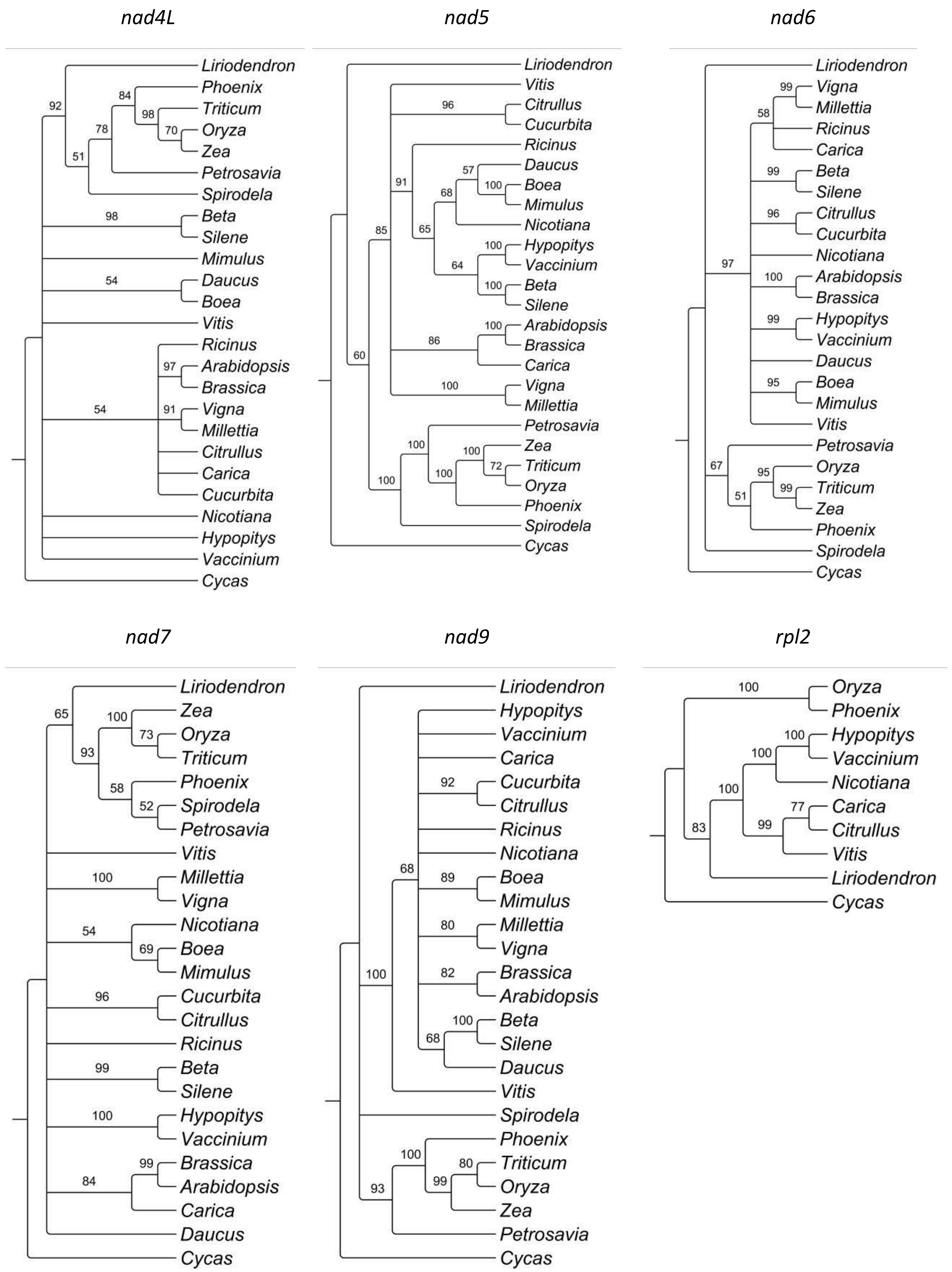
nad4L
Liriodendron
Phoenix
Triticum
Oryza
Zea
Petrosavia
Spirodela
Beta
Silene
Mimulus
Daucus
Boea
Vitis
Ricinus
Arabidopsis
Brassica
Vigna
Millettia
Citrullus
Carica
Cucurbita
Nicotiana
Hypopitys
Vaccinium
Cycas
nad5
Liriodendron
Vitis
Citrullus
Cucurbita
Ricinus
Daucus
Boea
Mimulus
Nicotiana
Hypopitys
Vaccinium
Beta
Silene
Arabidopsis
Brassica
Carica
Vigna
Millettia
Petrosavia
Zea
Triticum
Oryza
Phoenix
Spirodela
Cycas
nad6
Liriodendron
Vigna
Millettia
Ricinus
Carica
Beta
Silene
Citrullus
Cucurbita
Nicotiana
Arabidopsis
Brassica
Hypopitys
Vaccinium
Daucus
Boea
Mimulus
Vitis
Petrosavia
Oryza
Triticum
Zea
Phoenix
Spirodela
Cycas
nad7
Liriodendron
Zea
Oryza
Triticum
Phoenix
Spirodela
Petrosavia
Vitis
Millettia
Vigna
Nicotiana
Boea
Mimulus
Cucurbita
Citrullus
Ricinus
Beta
Silene
Hypopitys
Vaccinium
Brassica
Arabidopsis
Carica
Daucus
Cycas
nad9
Liriodendron
Hypopitys
Vaccinium
Carica
Cucurbita
Citrullus
Ricinus
Nicotiana
Boea
Mimulus
Millettia
Vigna
Brassica
Arabidopsis
Beta
Silene
Daucus
Vitis
Spirodela
Phoenix
Triticum
Oryza
Zea
Petrosavia
Cycas
rpl2
Oryza
Phoenix
Hypopitys
Vaccinium
Nicotiana
Carica
Citrullus
Vitis
Liriodendron
Cycas

rpl5

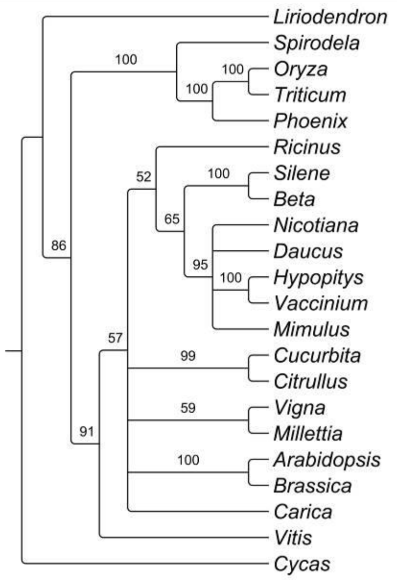

rpl10

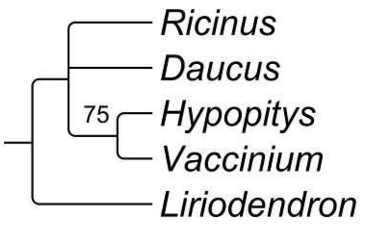

rpl16

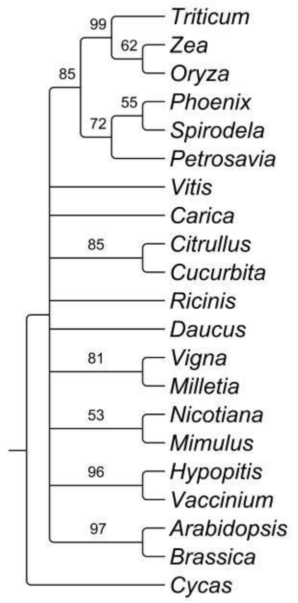

rps1

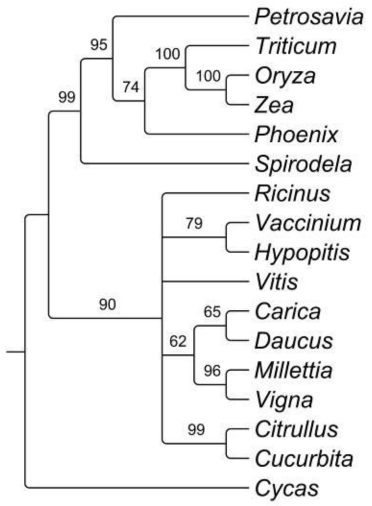

rps3

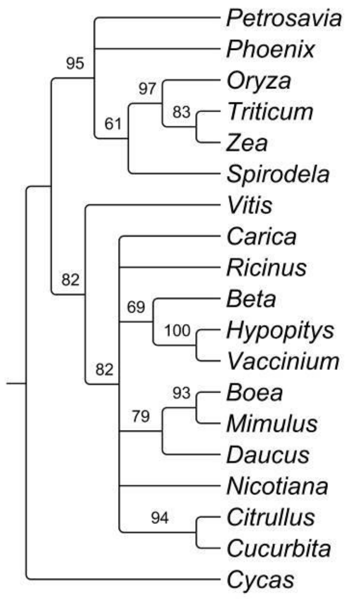

rps4

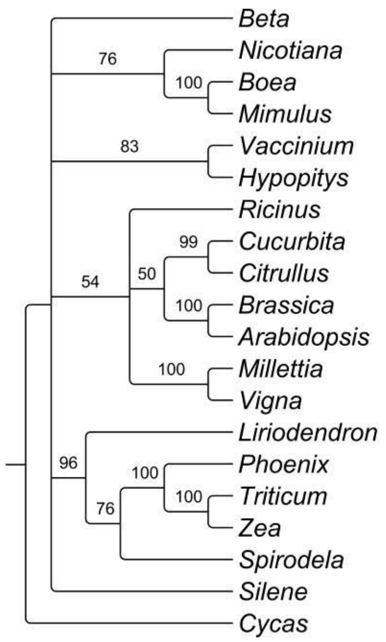

rps10

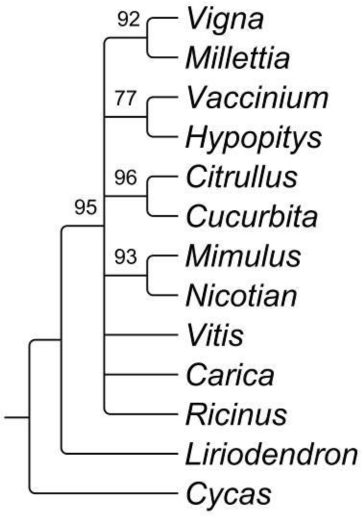

rps12

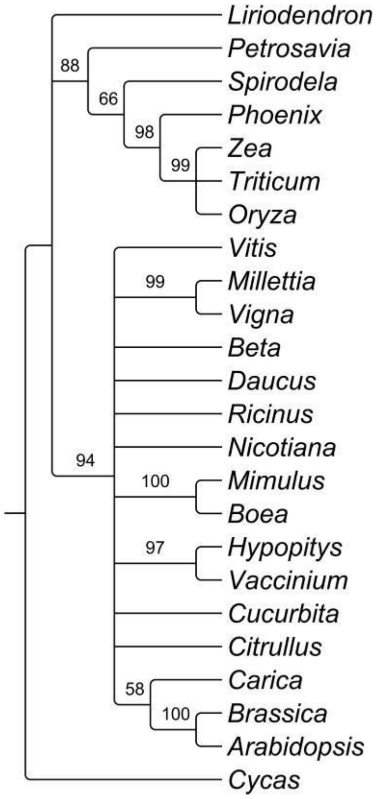

rps13

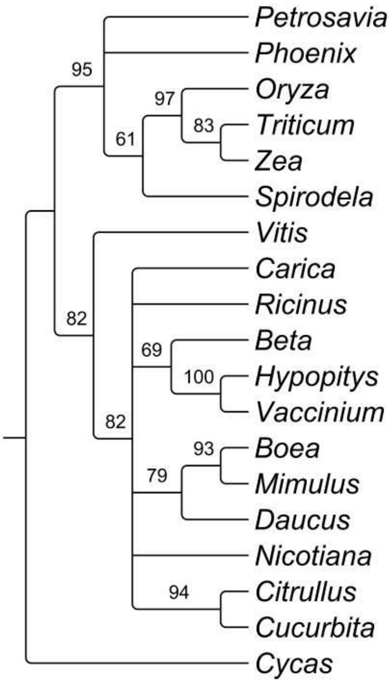

rps14

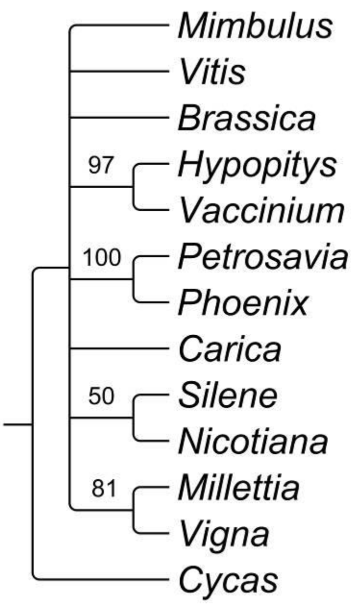

rps19

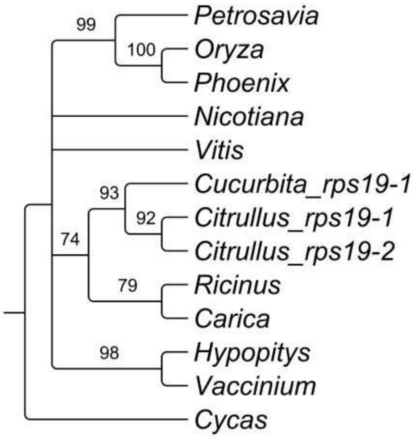

sdh4

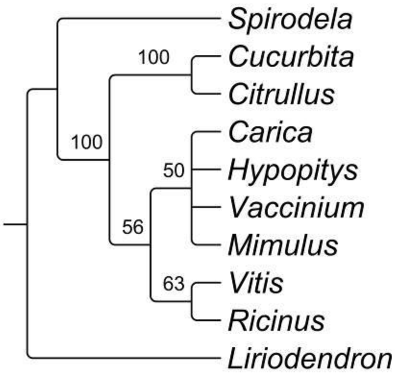

Supplement: Supplemental Information 5 [file peerj-08-9309-s005.pdf]
