# Supplementary material for: Mitochondrial genome of the nonphotosynthetic mycoheterotrophic plant Hypopitys monotropa, its structure, gene expression and RNA editing
Source: PeerJ. 2020 Jun 19;8:e9309. doi: 10.7717/peerj.9309 (PMC7307570; doi:10.7717/peerj.9309)
Supplement: Supplemental Information 8 [file peerj-08-9309-s008.pdf]

A

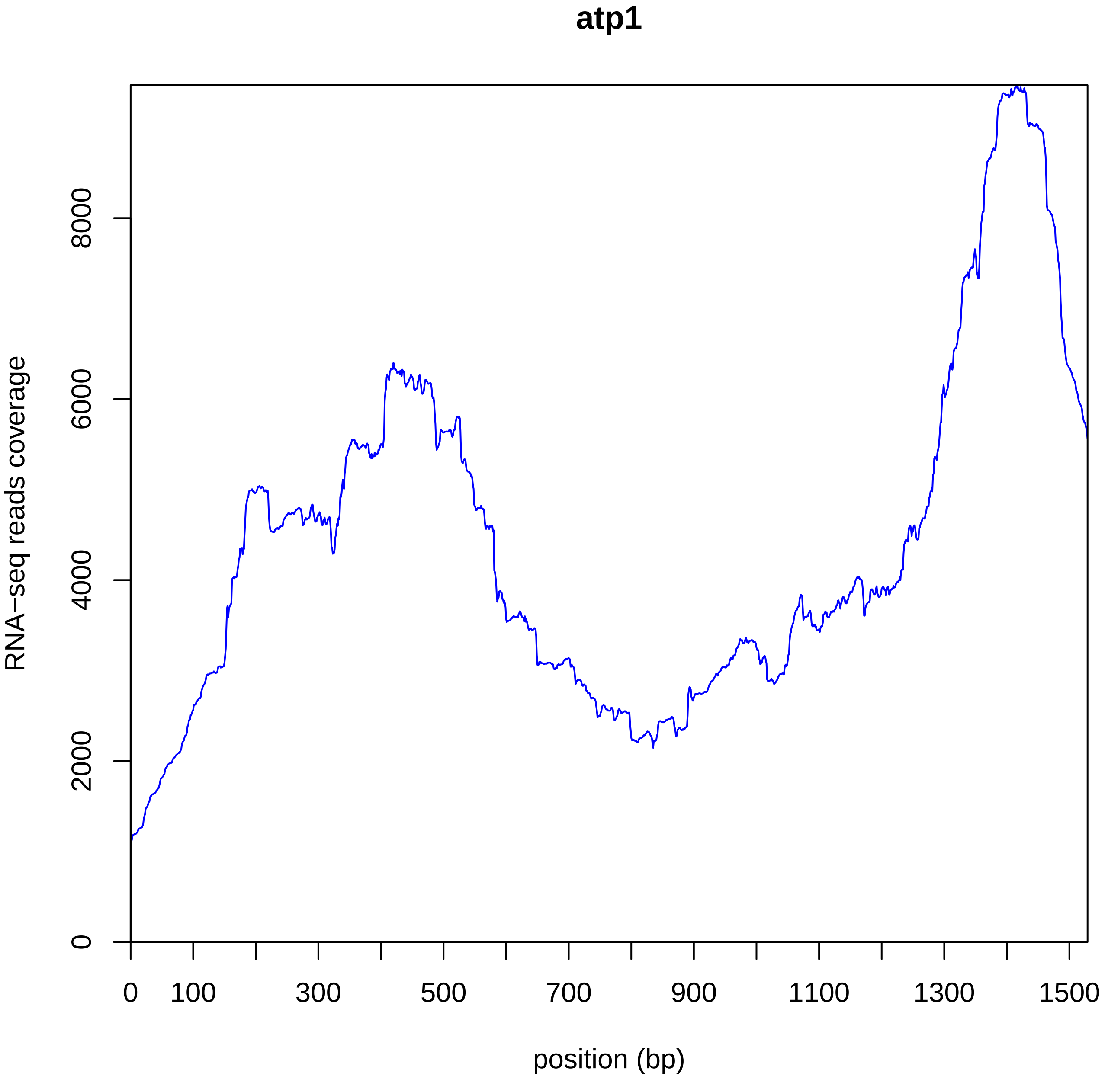
atp1
RNA−seq reads coverage
0
2000
4000
6000
8000
0
100
300
500
700
900
1100
1300
1500
position (bp)

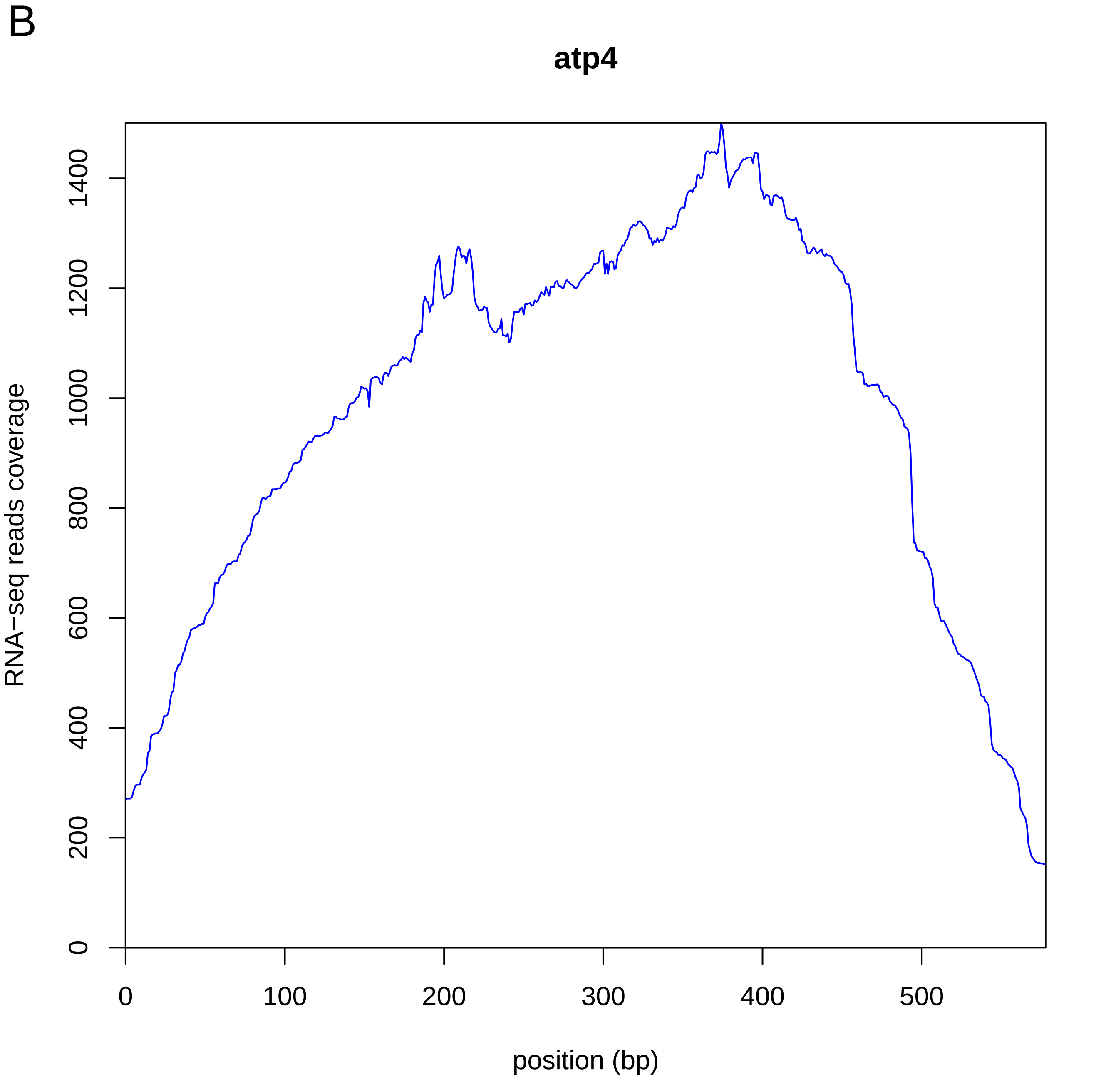
B
atp4
RNA−seq reads coverage
0
200
400
600
800
1000
1200
1400
0
100
200
300
400
500
position (bp)

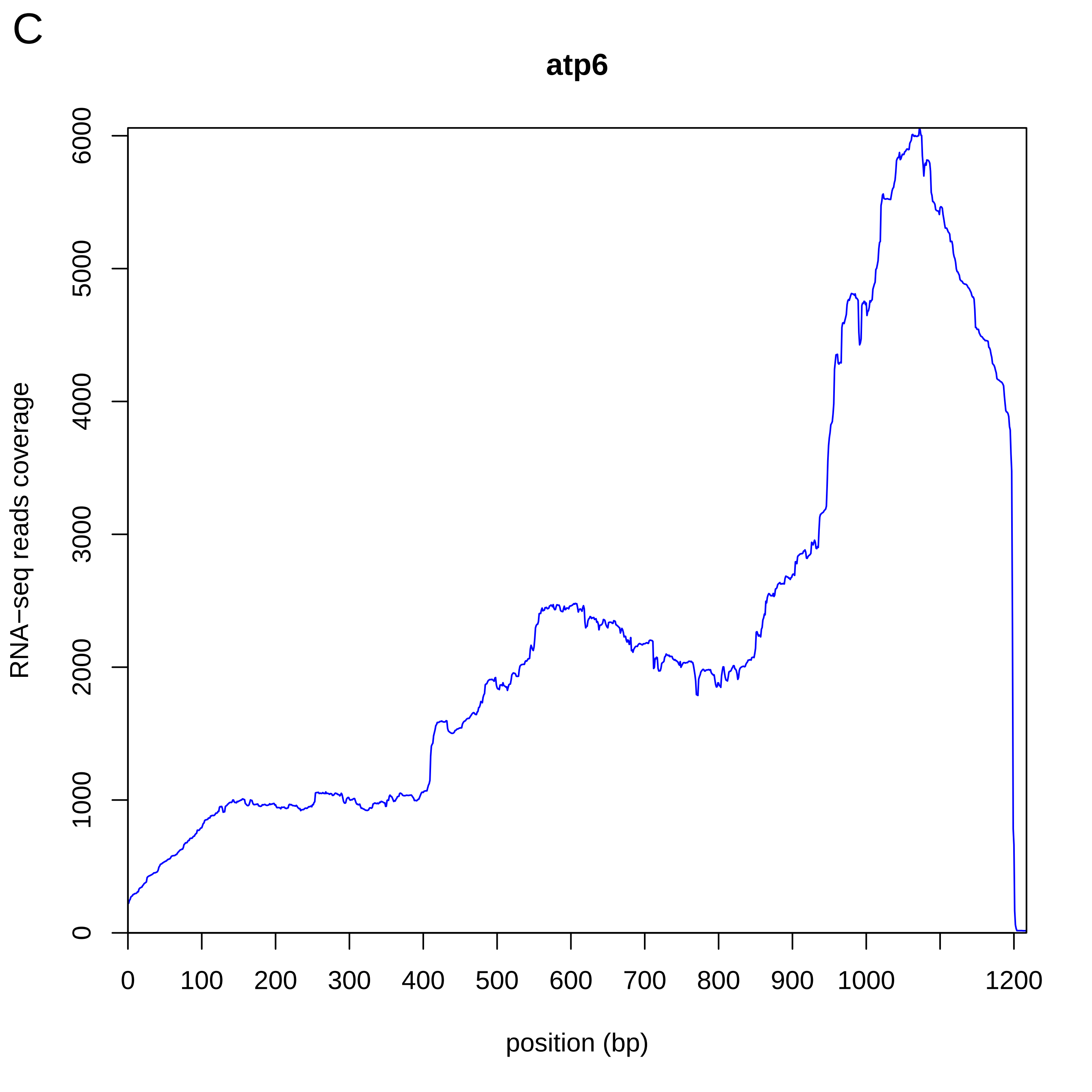

C
atp6
RNA−seq reads coverage
0
1000
2000
3000
4000
5000
6000
0
100
200
300
400
500
600
700
800
900
1000
1200
position (bp)

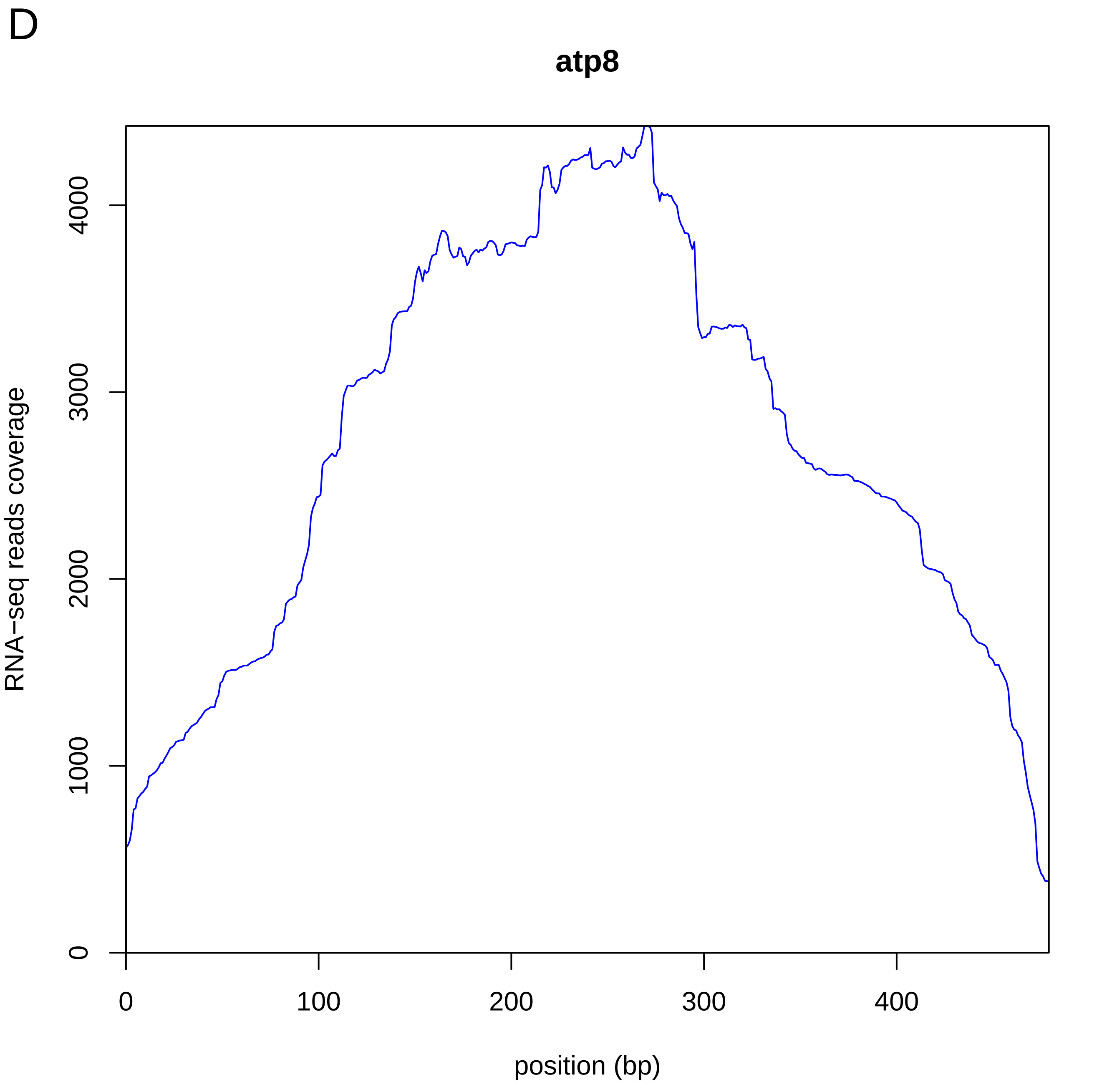

D
atp8
RNA−seq reads coverage
0
1000
2000
3000
4000
0
100
200
300
400
position (bp)

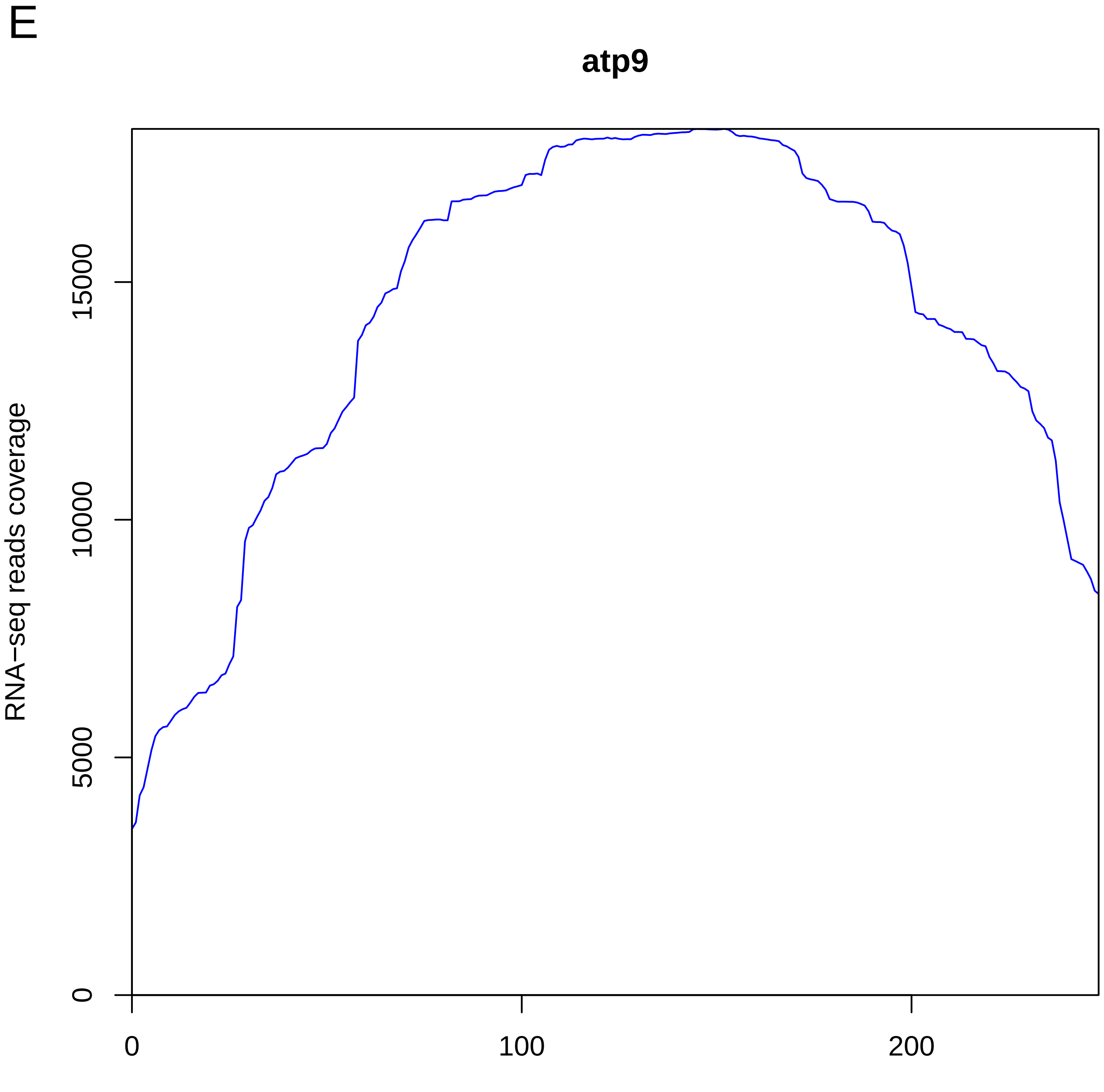
E
atp9
RNA−seq reads coverage
0
5000
10000
15000
0
100
200
position (bp)

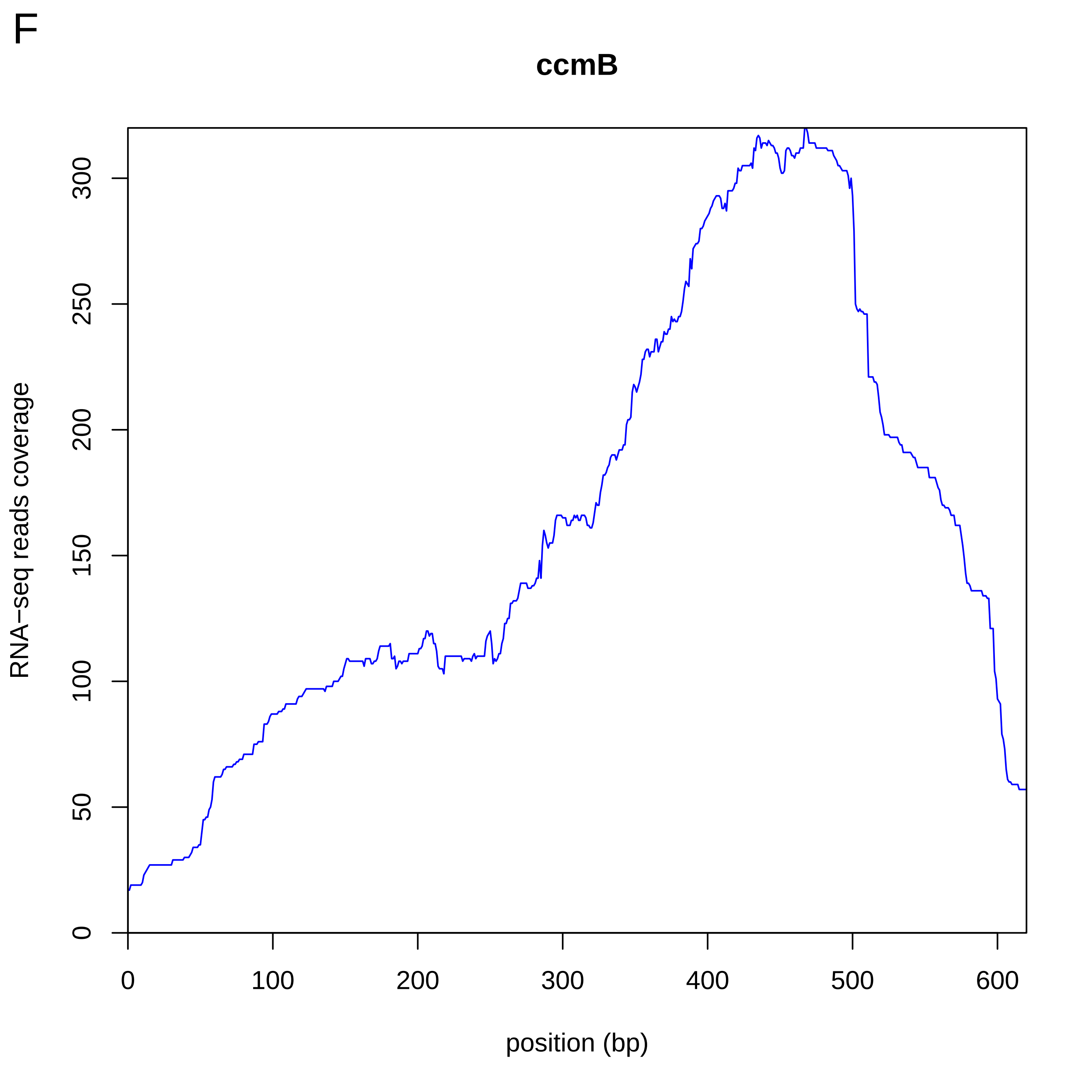
F
ccmB
RNA−seq reads coverage
position (bp)
0
50
100
150
200
250
300
0
100
200
300
400
500
600

G

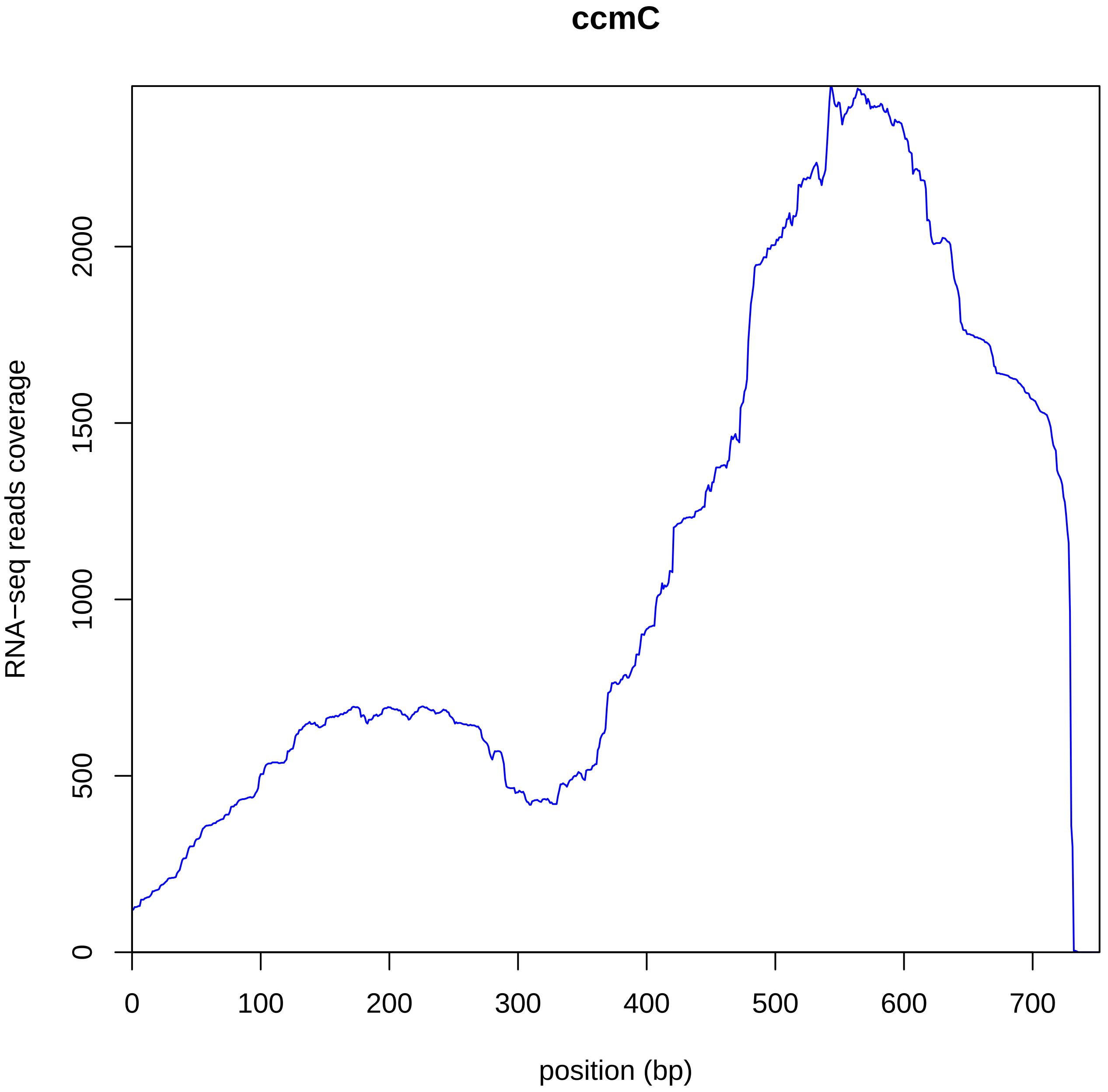

ccmC
RNA−seq reads coverage
position (bp)
0
500
1000
1500
2000
0
100
200
300
400
500
600
700

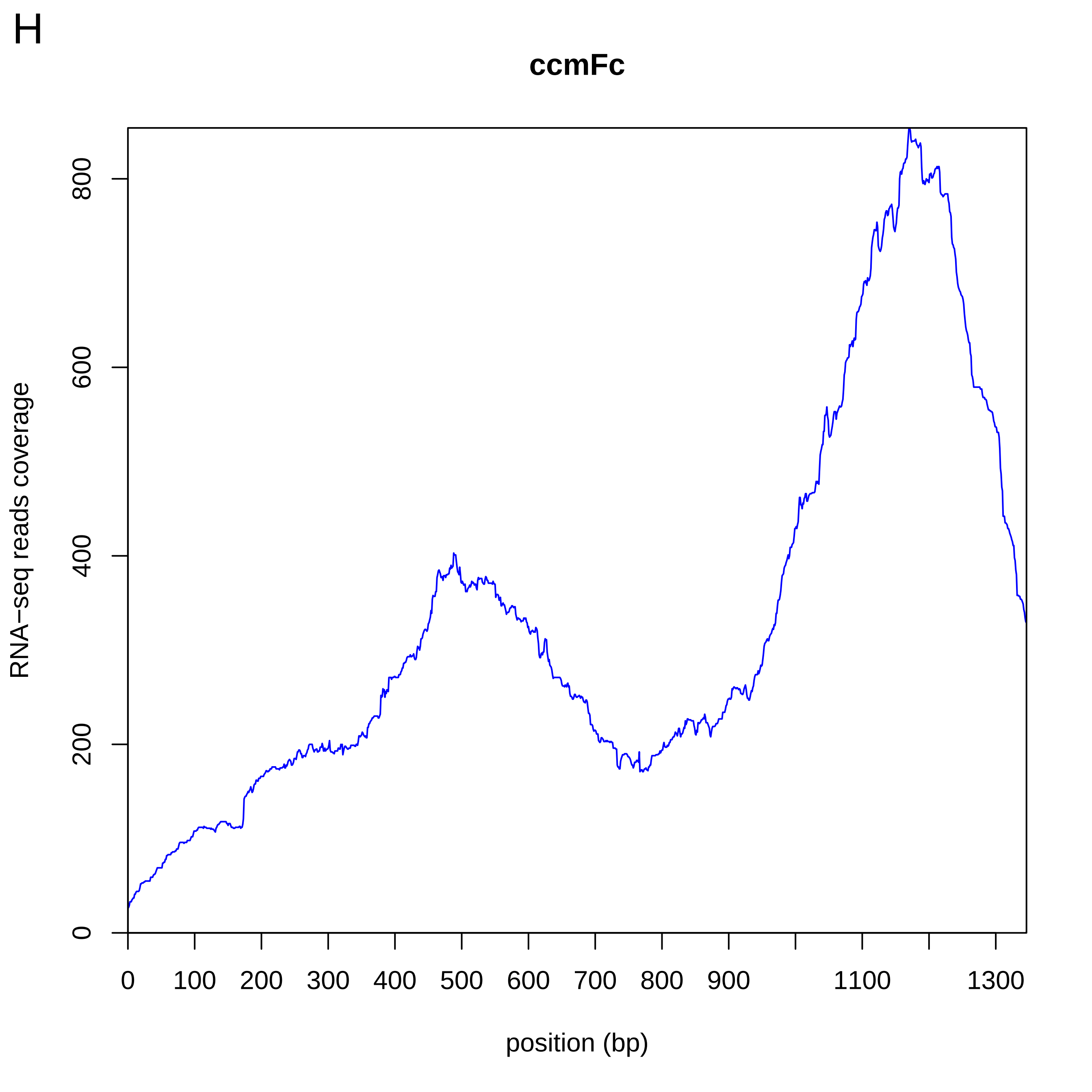

H
ccmFc
RNA−seq reads coverage
0
200
400
600
800
0
100
200
300
400
500
600
700
800
900
1100
1300
position (bp)

I

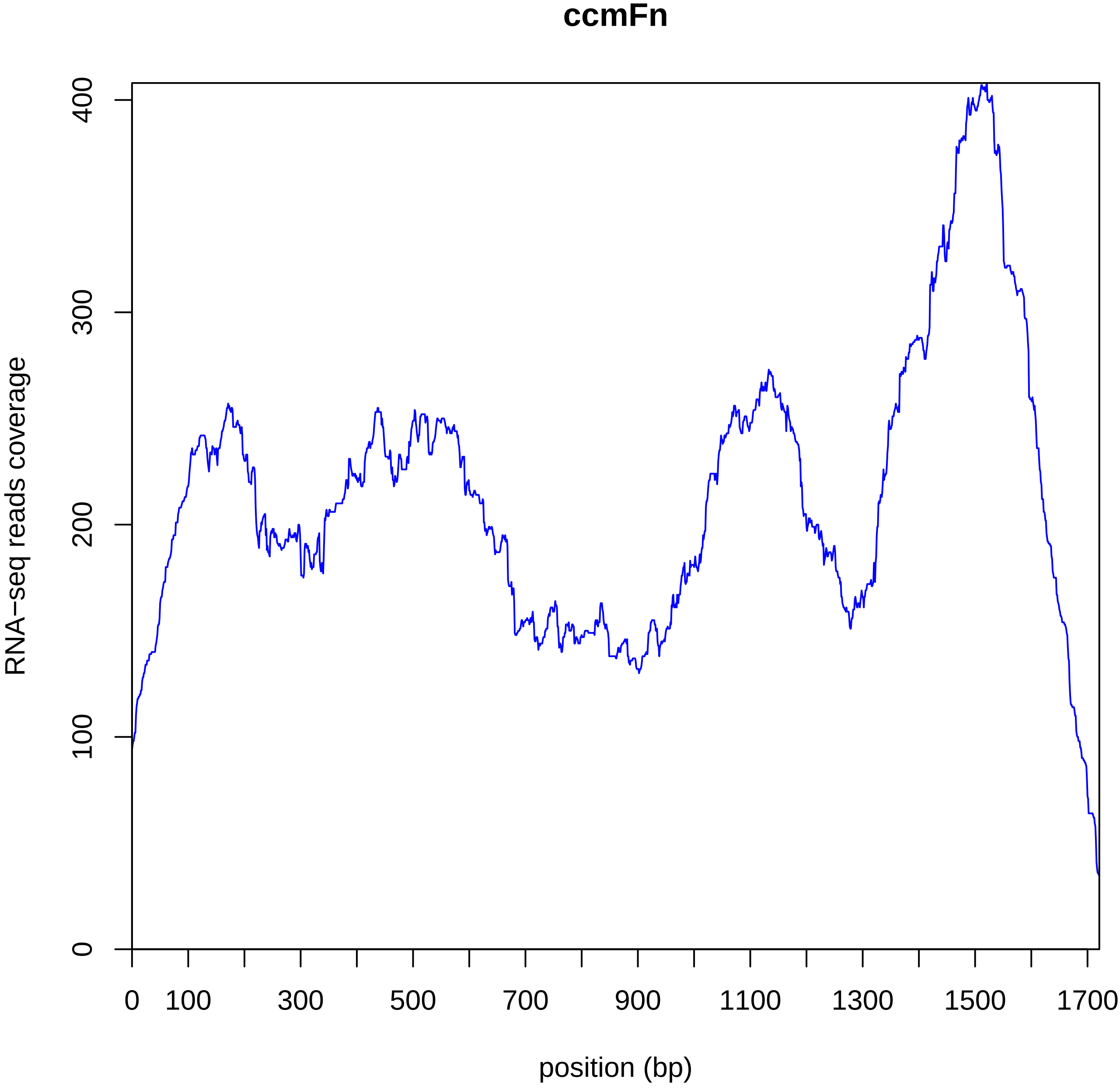

ccmFn
RNA−seq reads coverage
position (bp)
0
100
200
300
400
0
100
300
500
700
900
1100
1300
1500
1700

J

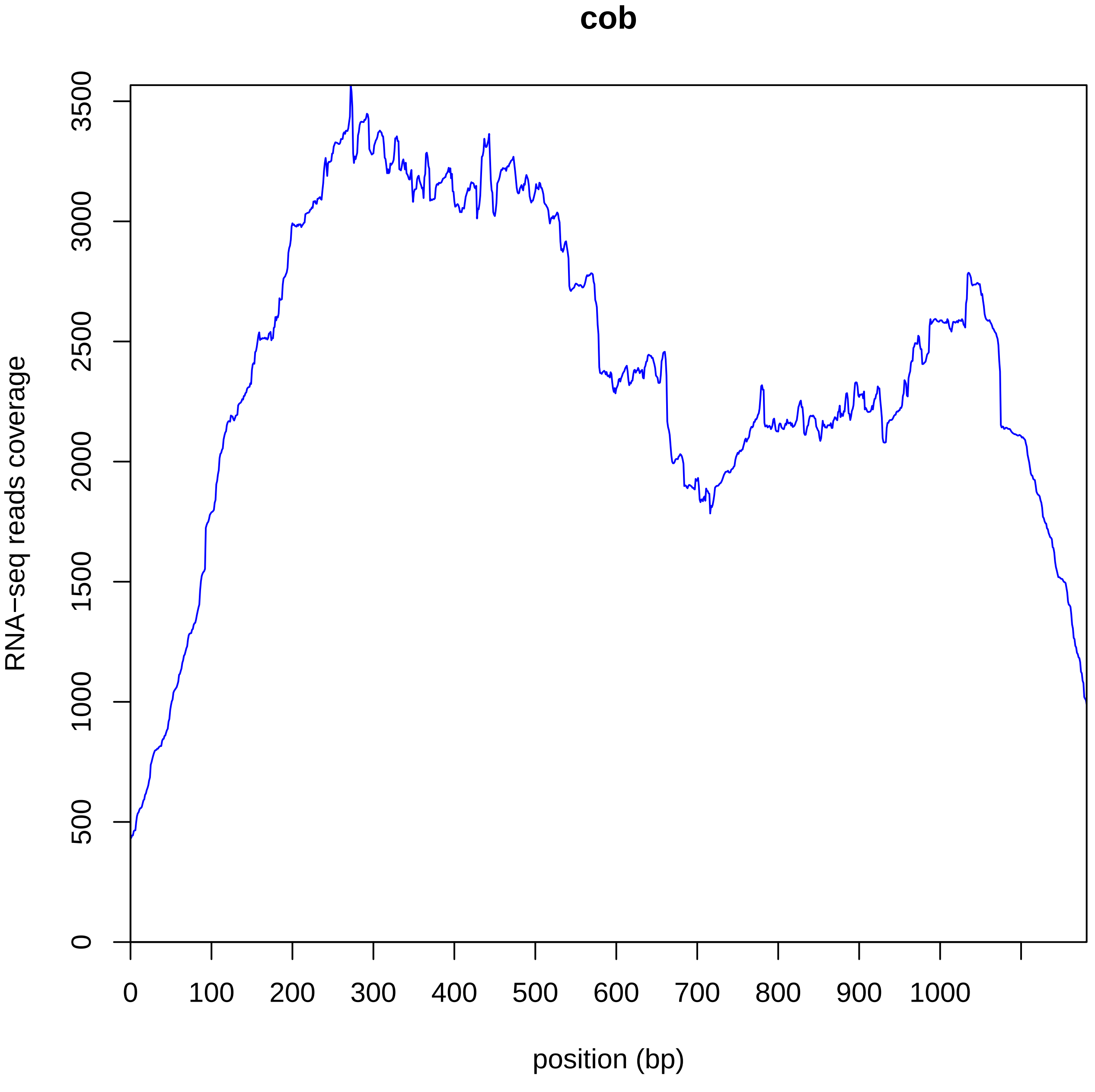
cob
RNA−seq reads coverage
0
500
1000
1500
2000
2500
3000
3500
0
100
200
300
400
500
600
700
800
900
1000
position (bp)

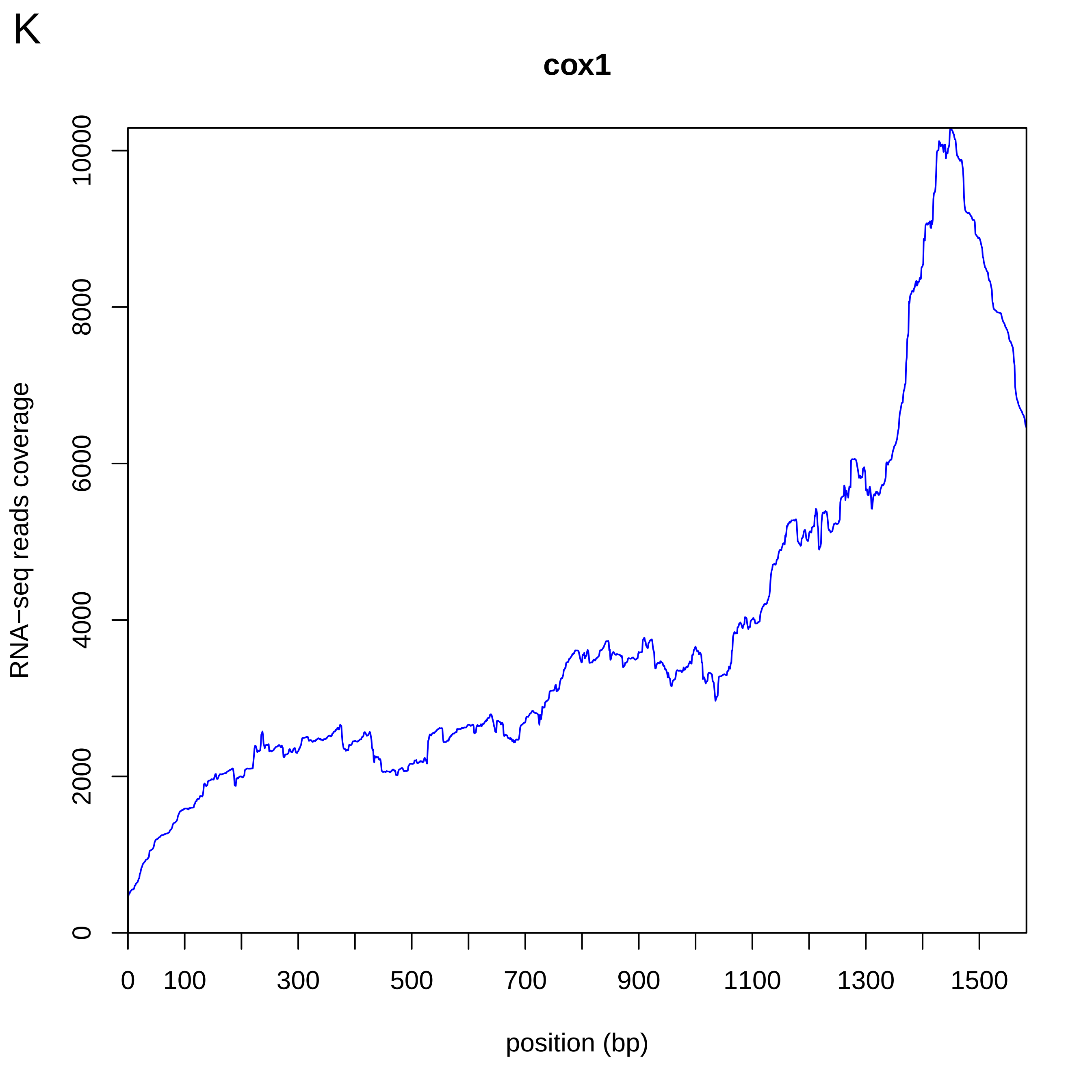
K
cox1
RNA−seq reads coverage
0
2000
4000
6000
8000
10000
0
100
300
500
700
900
1100
1300
1500
position (bp)

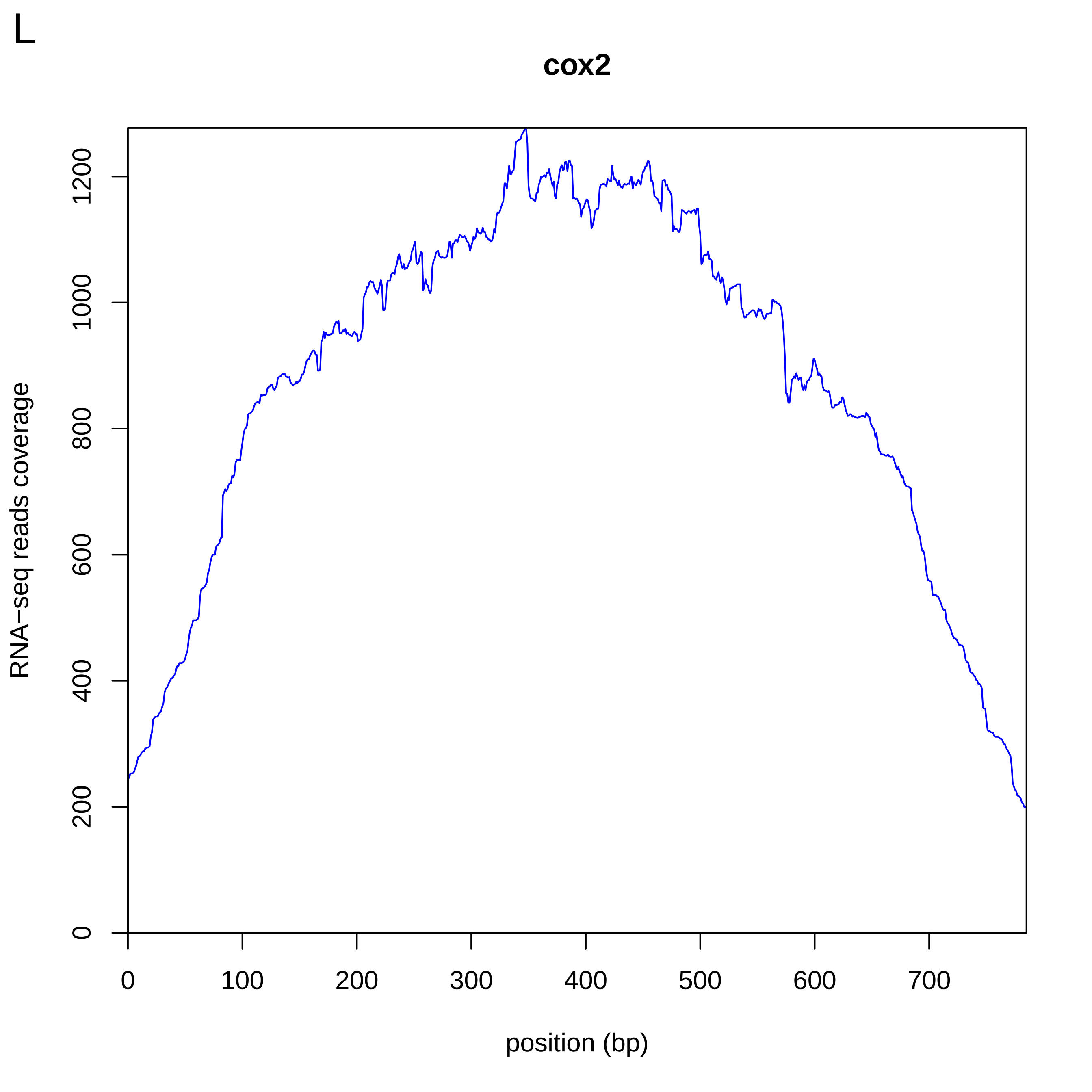
L
cox2
RNA−seq reads coverage
0
200
400
600
800
1000
1200
0
100
200
300
400
500
600
700
position (bp)

M

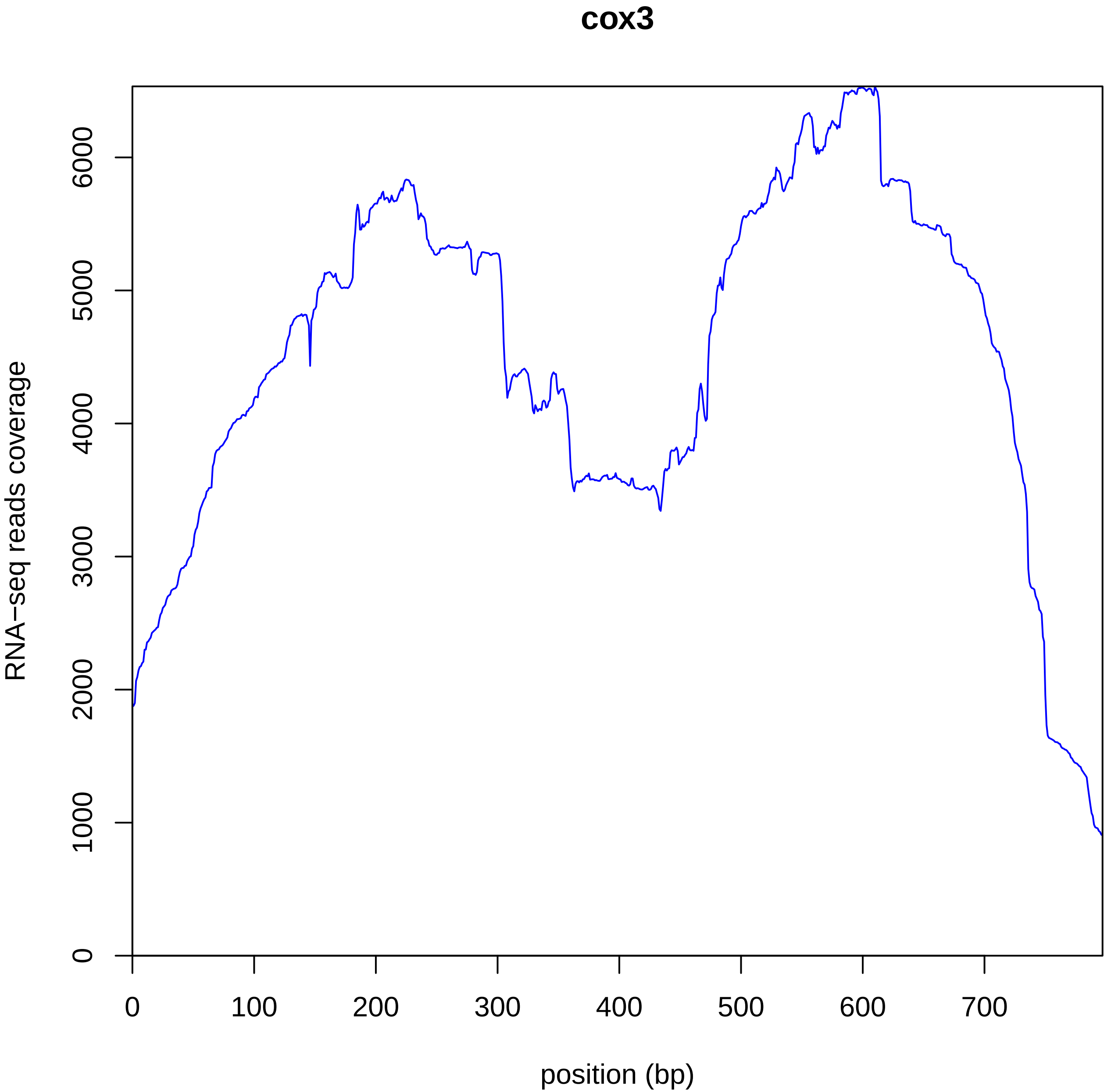
cox3
RNA−seq reads coverage
0
1000
2000
3000
4000
5000
6000
0
100
200
300
400
500
600
700
position (bp)

N

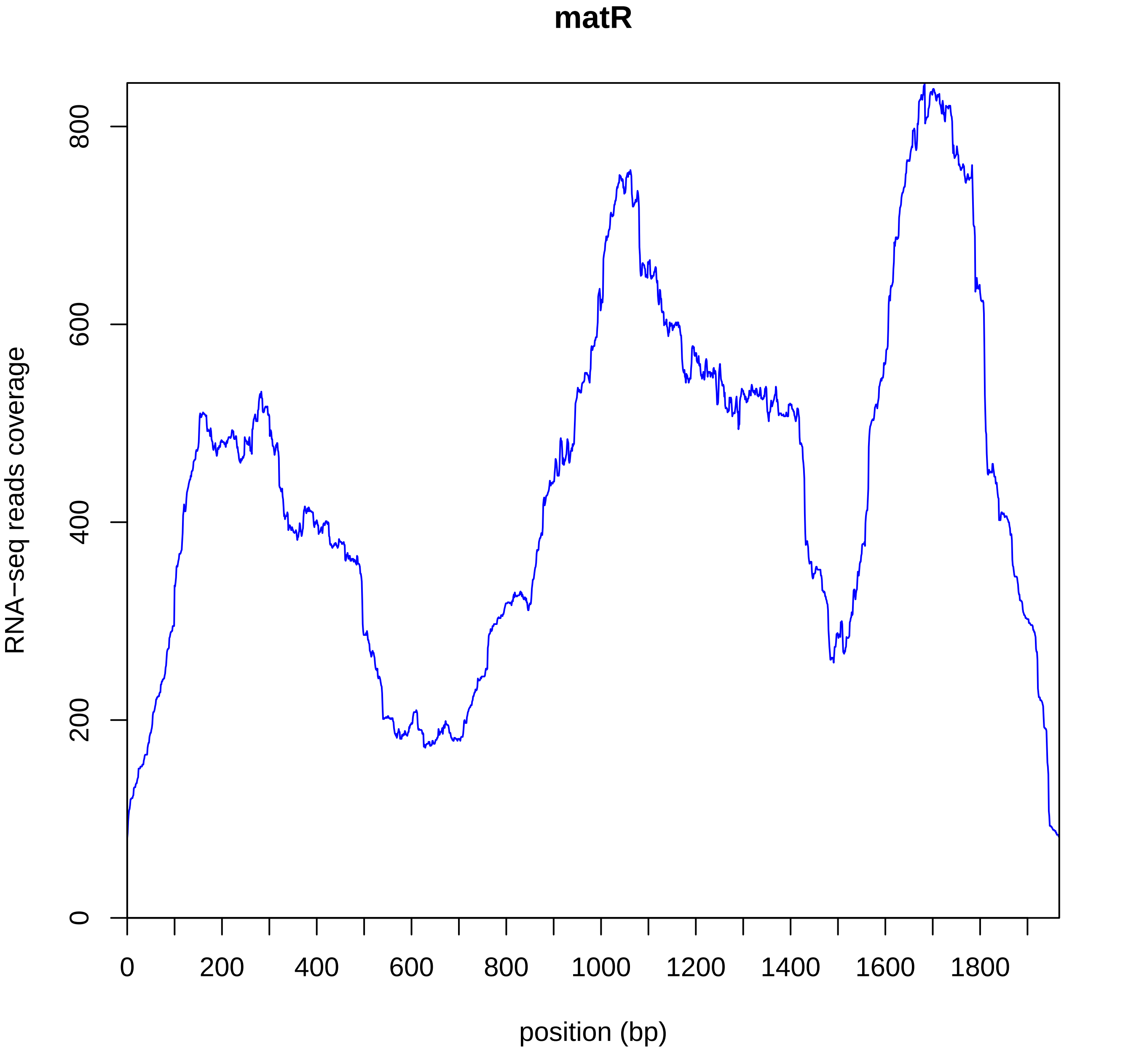
matR
RNA−seq reads coverage
0
200
400
600
800
0
200
400
600
800
1000
1200
1400
1600
1800
position (bp)

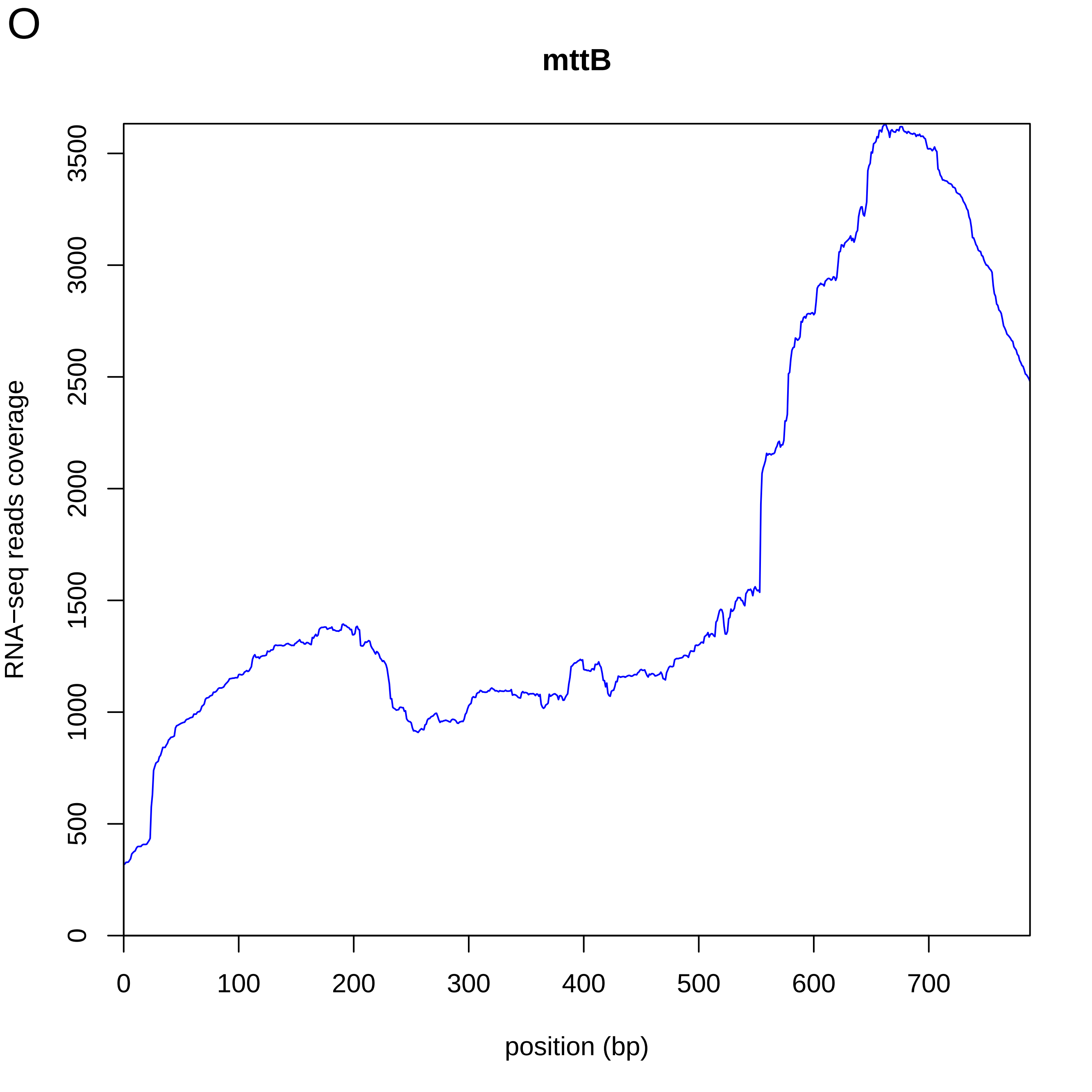
O
mttB
RNA−seq reads coverage
0
500
1000
1500
2000
2500
3000
3500
0
100
200
300
400
500
600
700
position (bp)

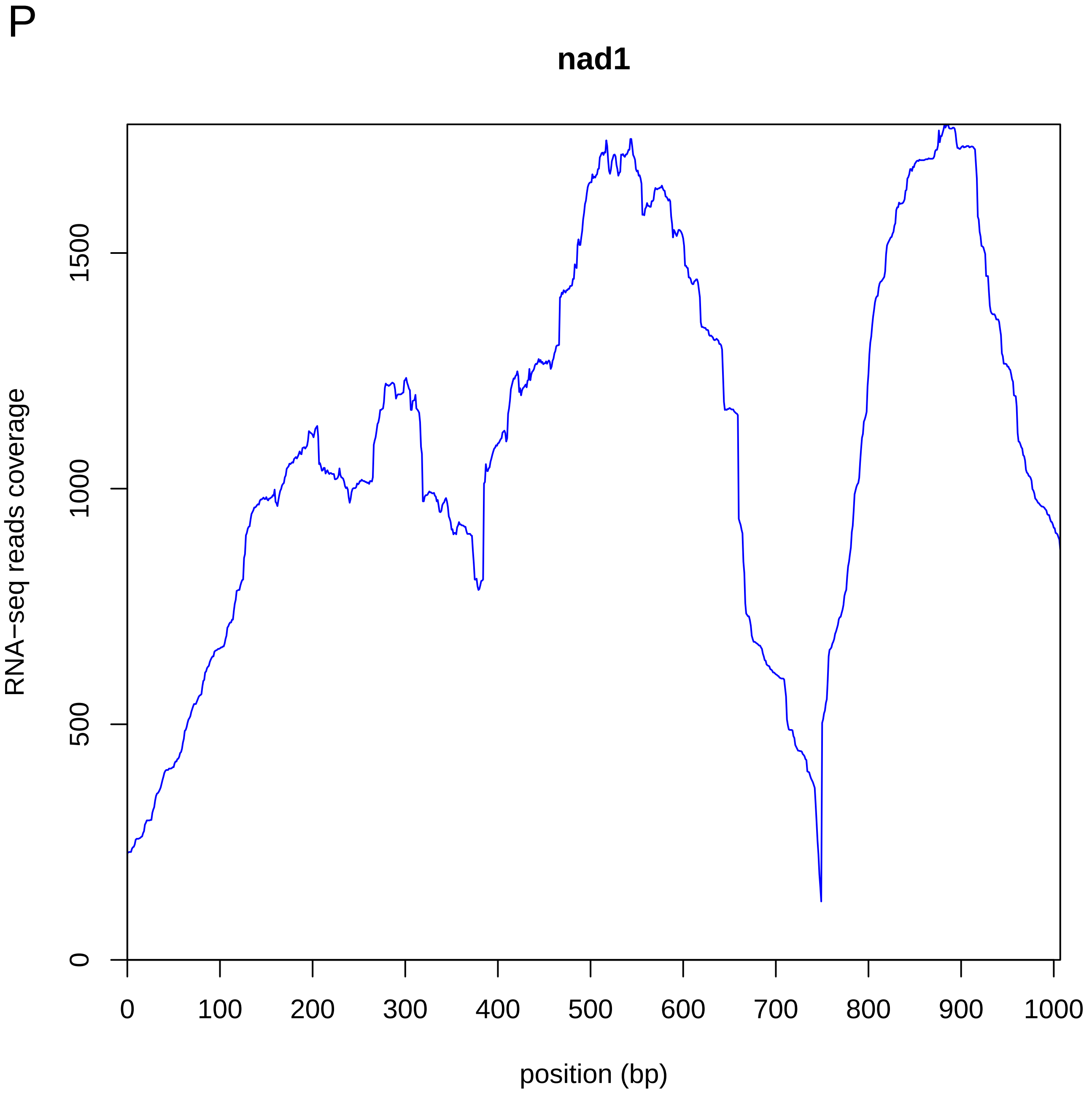
P
nad1
RNA−seq reads coverage
0
500
1000
1500
0
100
200
300
400
500
600
700
800
900
1000
position (bp)

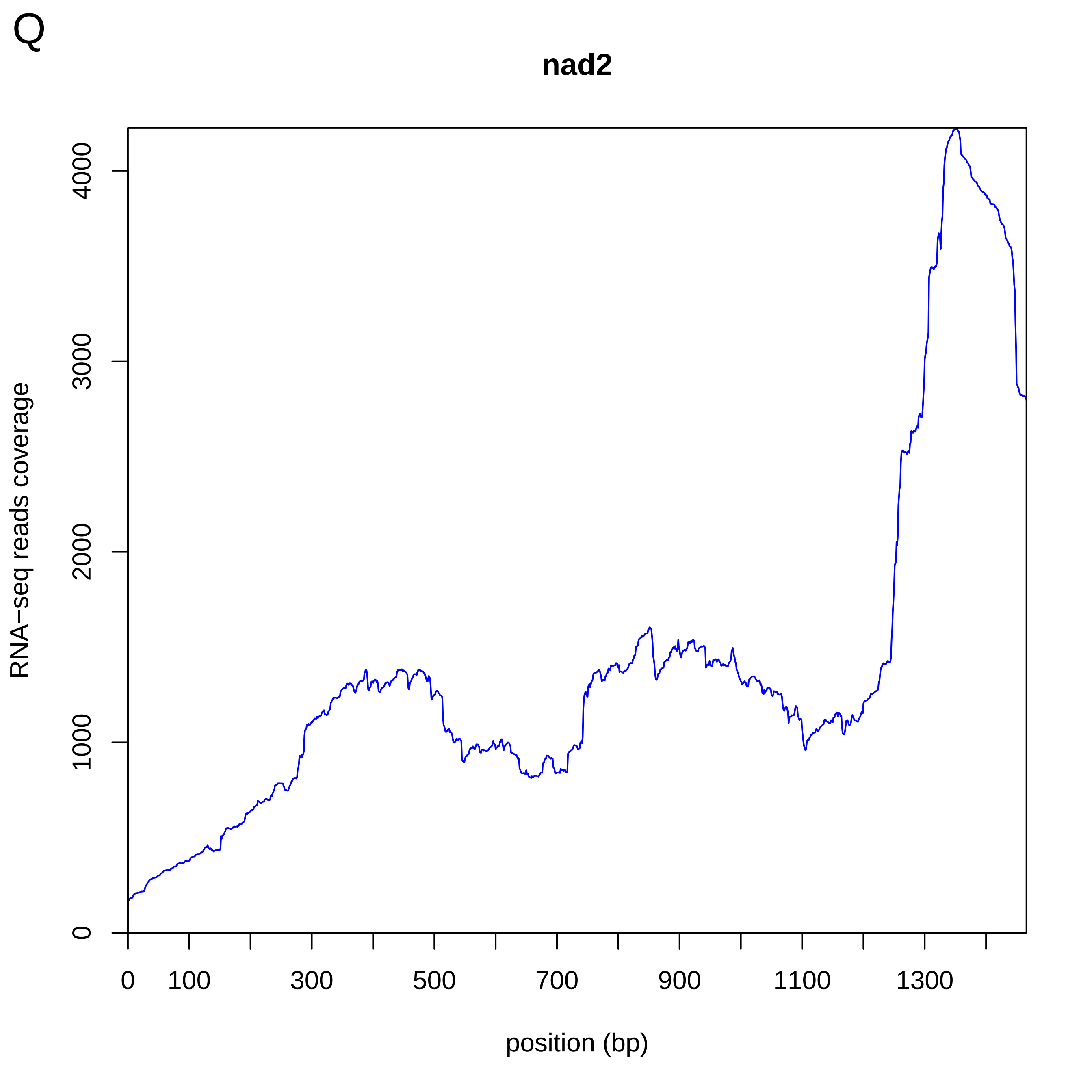
Q
nad2
RNA−seq reads coverage
0
1000
2000
3000
4000
0
100
300
500
700
900
1100
1300
position (bp)

R

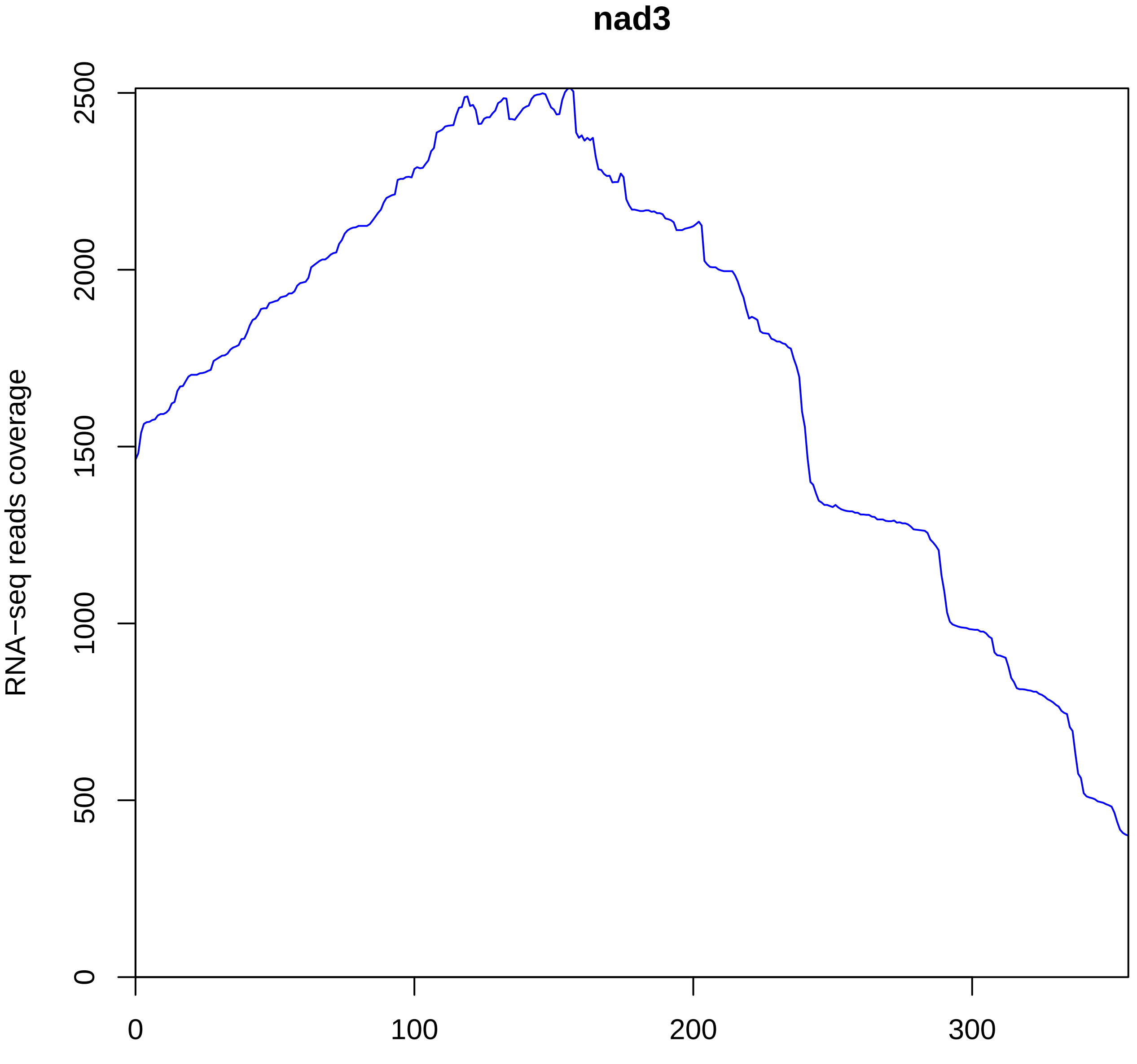
nad3
RNA−seq reads coverage
2500
2000
1500
1000
500
0
0
100
200
300
position (bp)

S

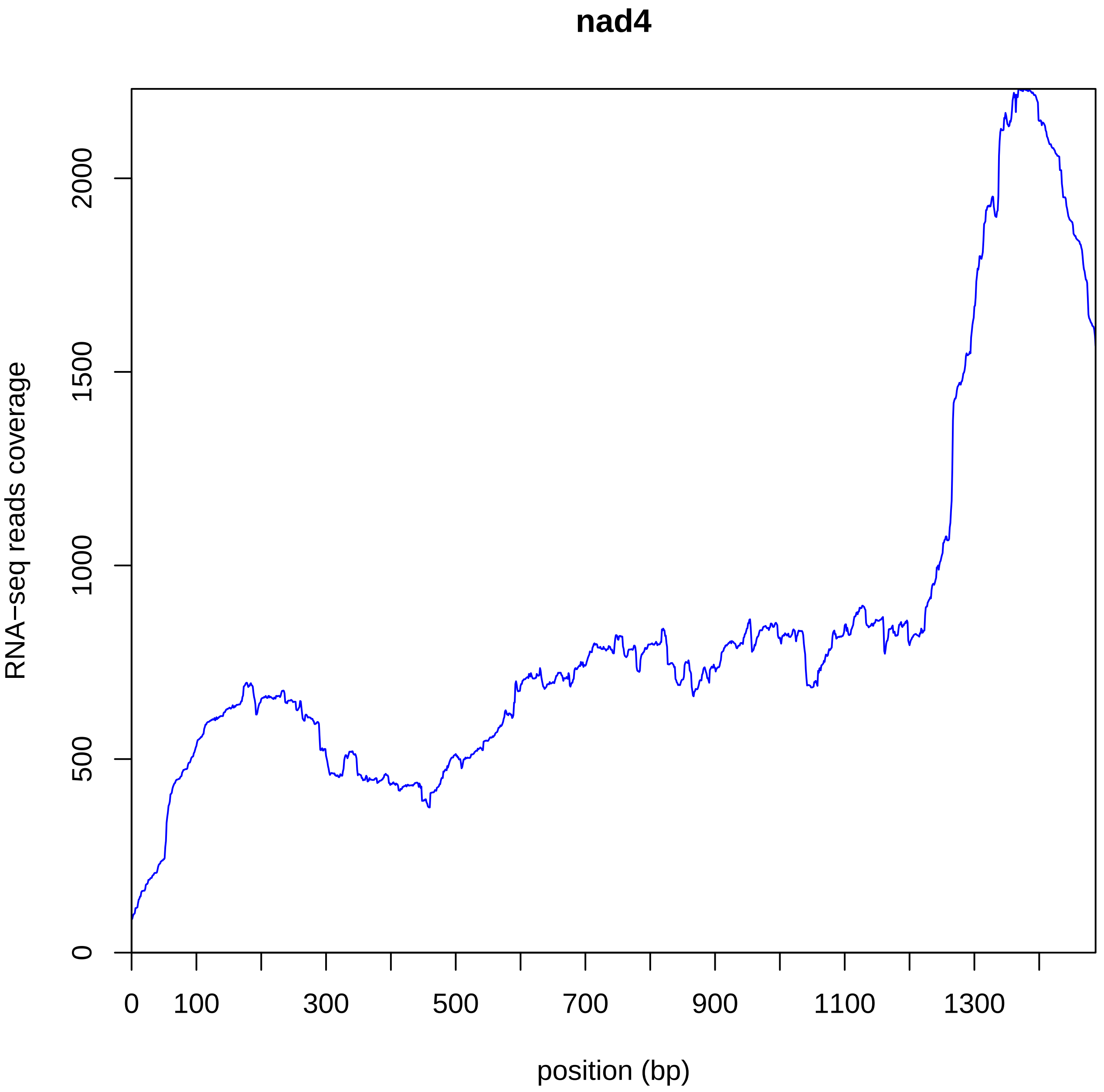
nad4
RNA−seq reads coverage
0
500
1000
1500
2000
0
100
300
500
700
900
1100
1300
position (bp)

T

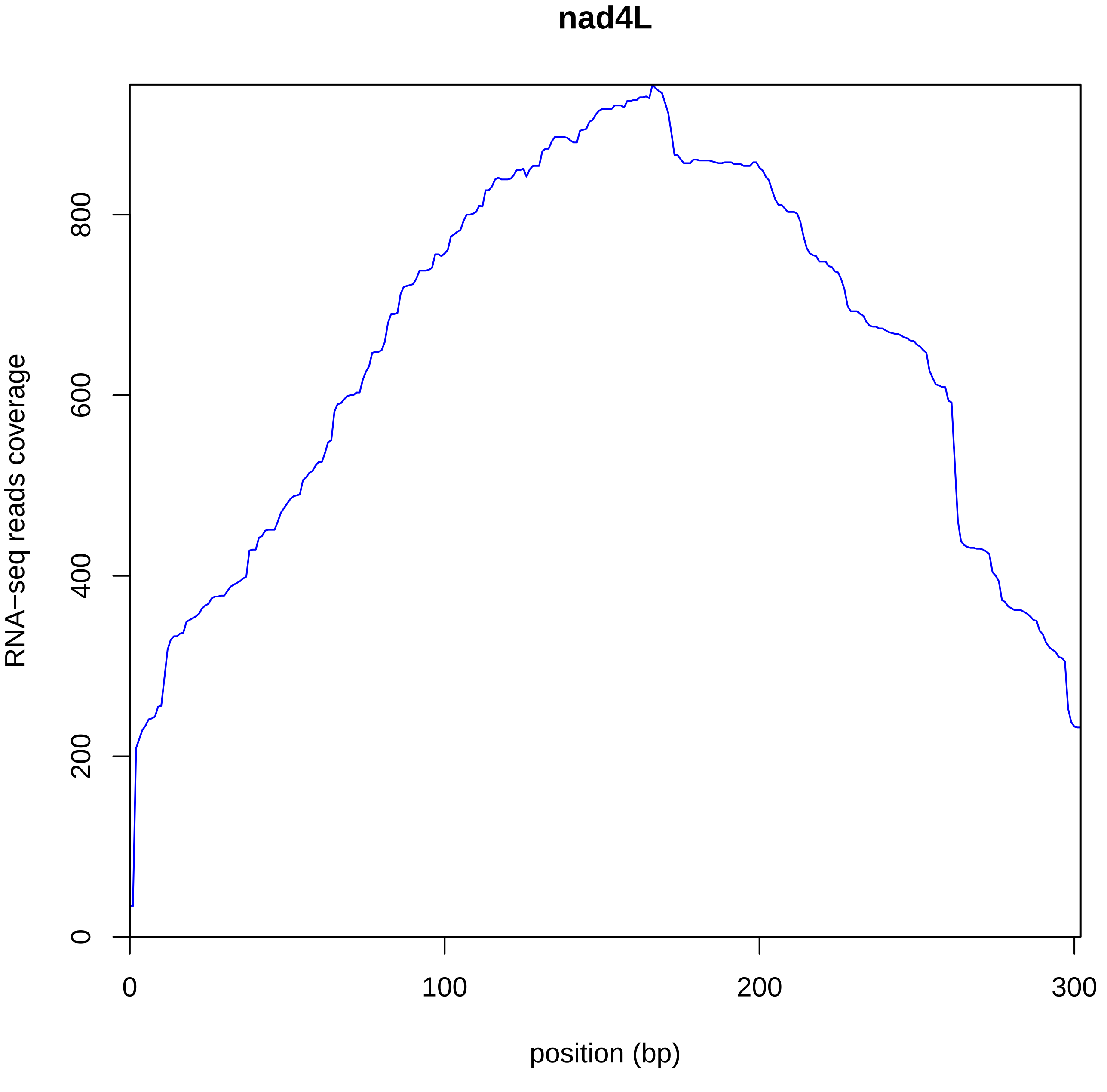

nad4L
RNA−seq reads coverage
0
200
400
600
800
0
100
200
300
position (bp)

U

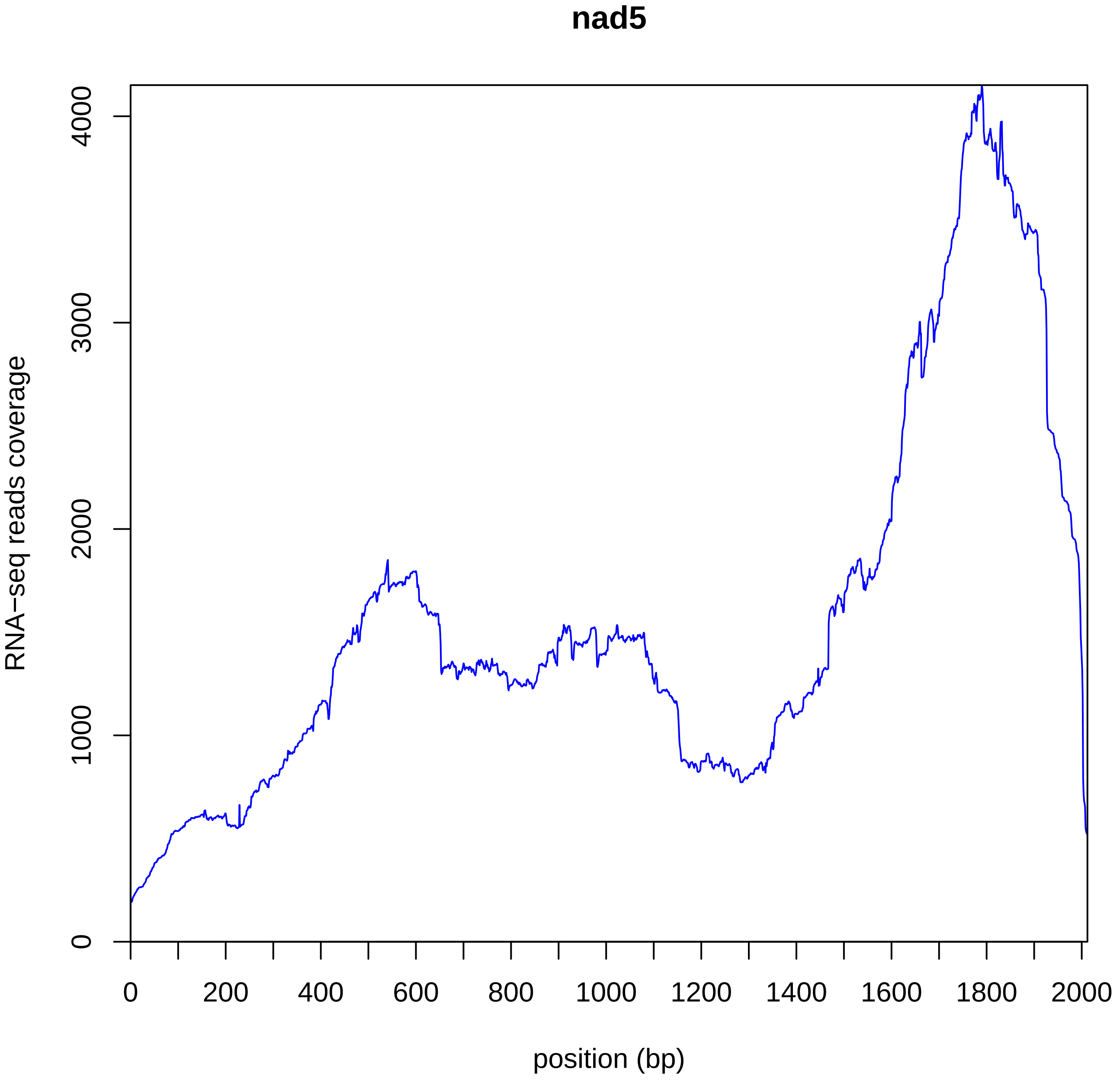

nad5
4000
3000
2000
1000
0
RNA−seq reads coverage
0
200
400
600
800
1000
1200
1400
1600
1800
2000
position (bp)

V

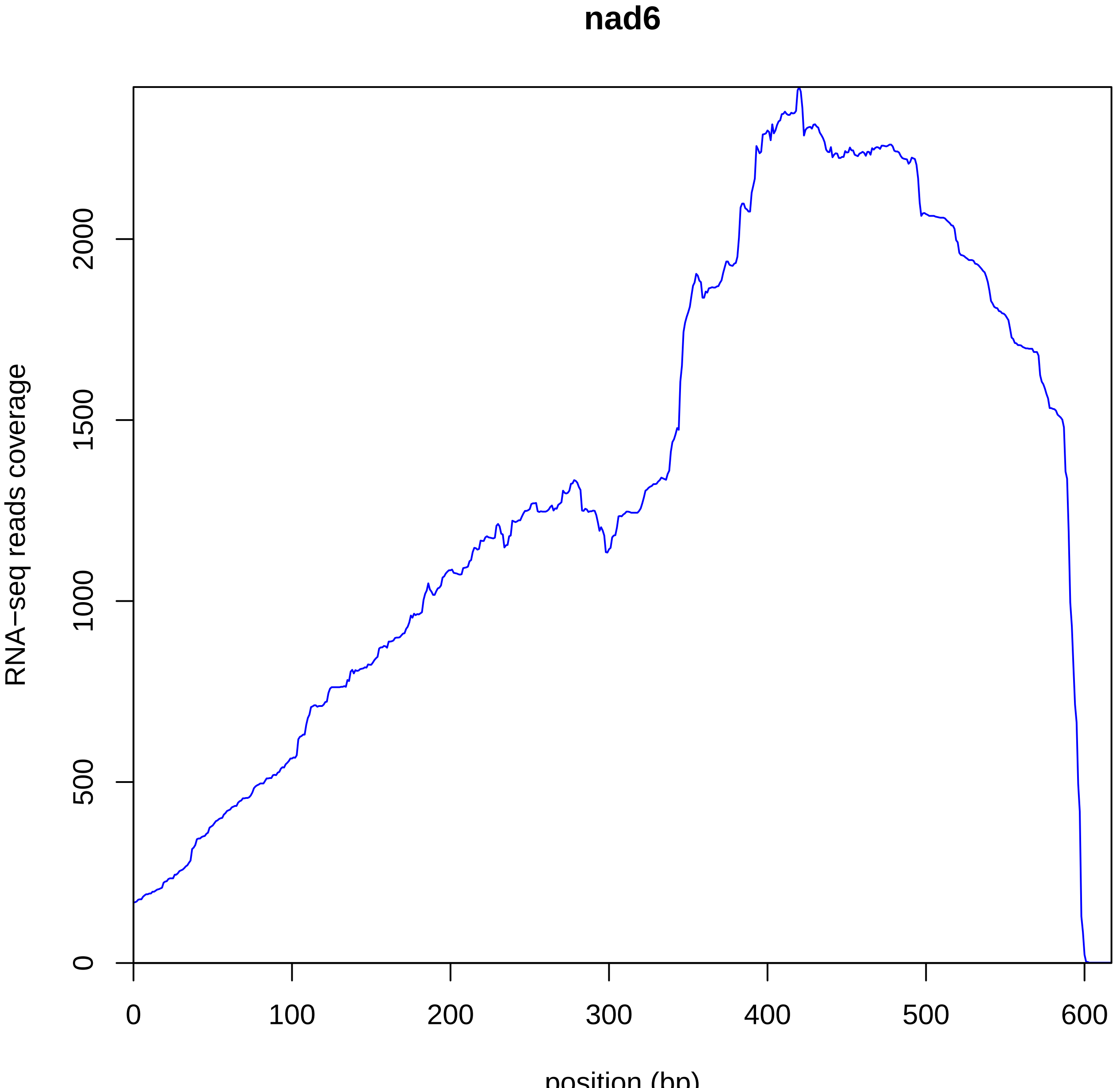
nad6
RNA−seq reads coverage
0
500
1000
1500
2000
0
100
200
300
400
500
600
position (bp)

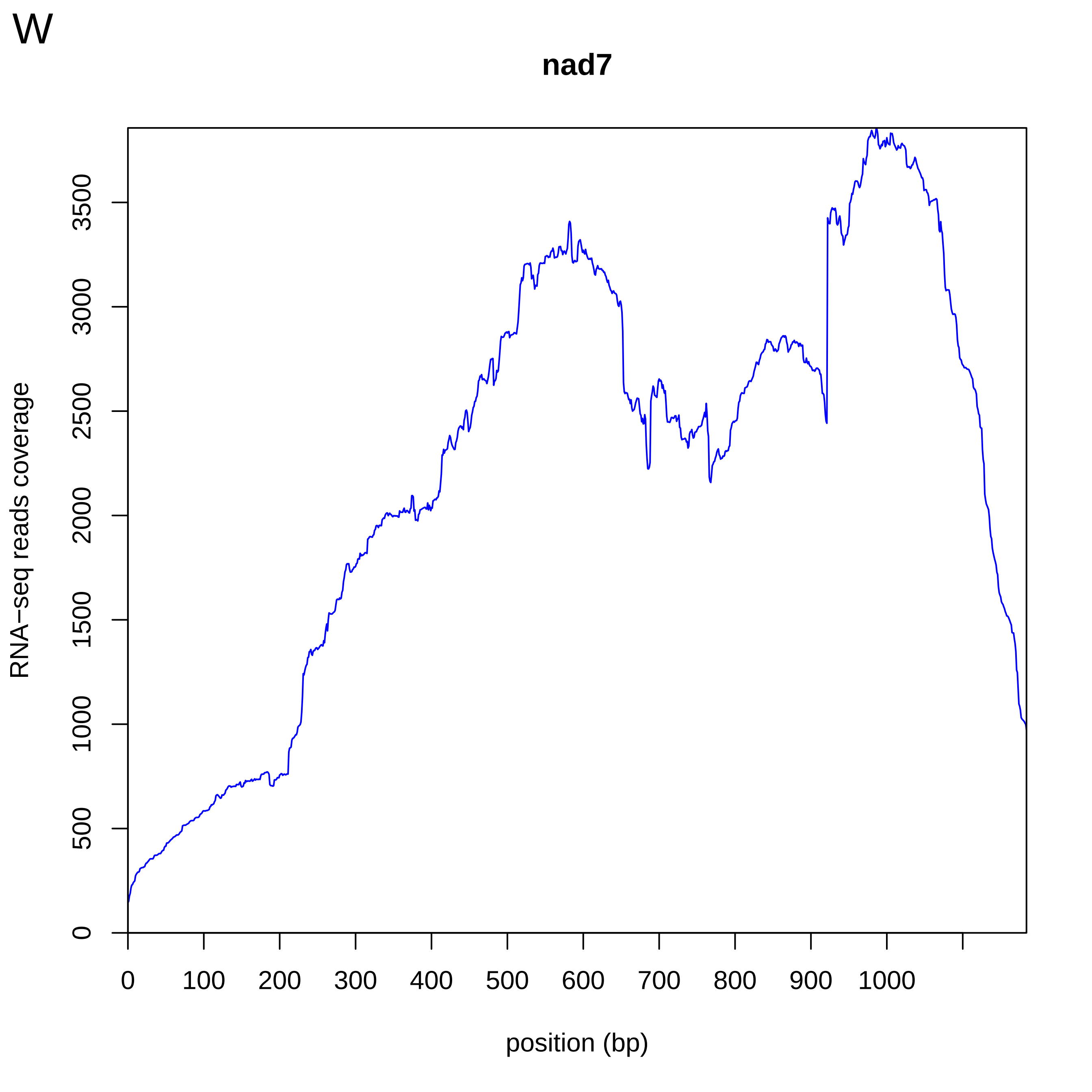
W
nad7
RNA−seq reads coverage
0
500
1000
1500
2000
2500
3000
3500
position (bp)
0
100
200
300
400
500
600
700
800
900
1000

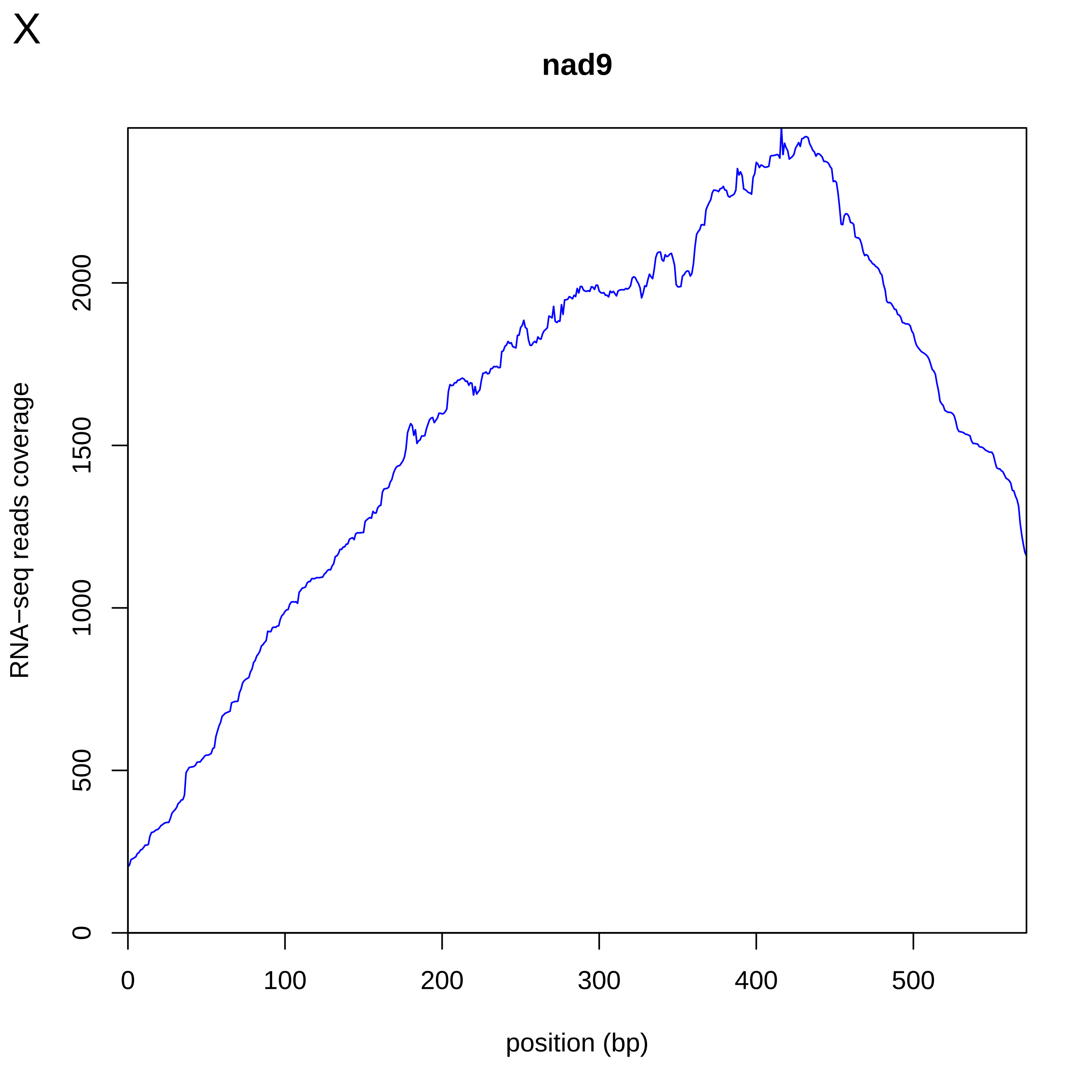
X
nad9
RNA−seq reads coverage
0
500
1000
1500
2000
0
100
200
300
400
500
position (bp)

Y

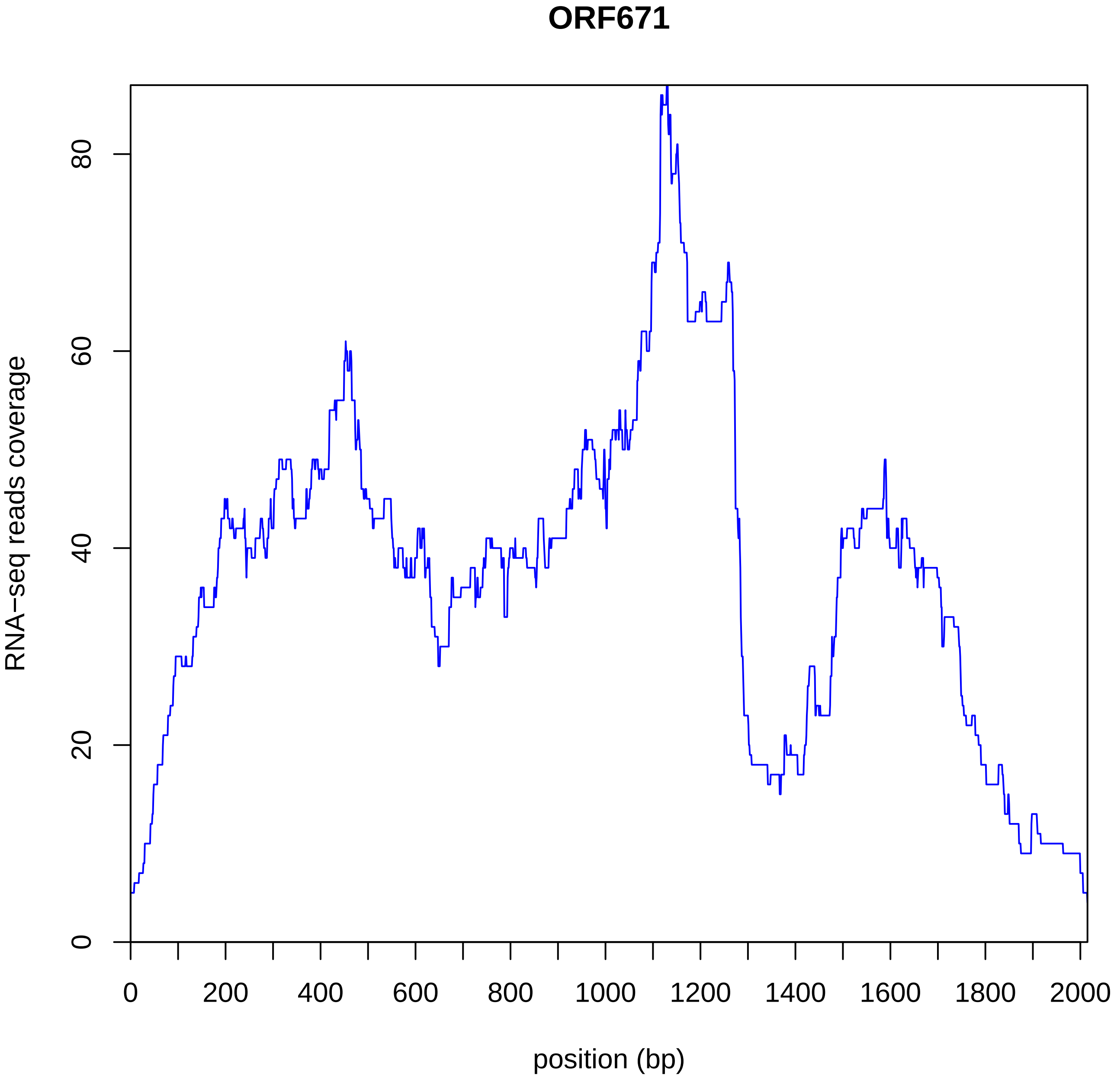

ORF671
RNA−seq reads coverage
0
20
40
60
80
0
200
400
600
800
1000
1200
1400
1600
1800
2000
position (bp)

Z

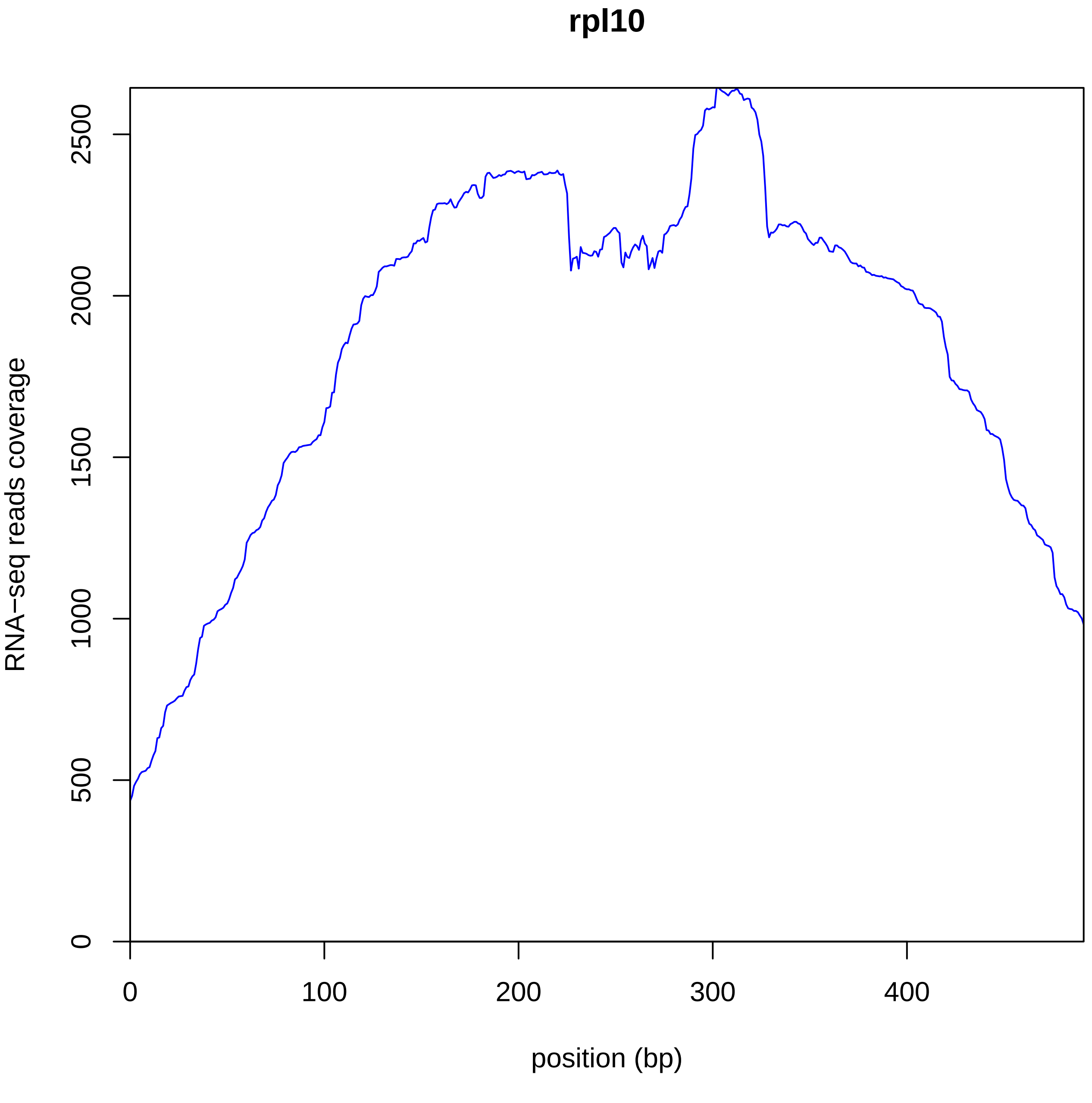
rpl10
RNA−seq reads coverage
0
500
1000
1500
2000
2500
0
100
200
300
400
position (bp)

AA

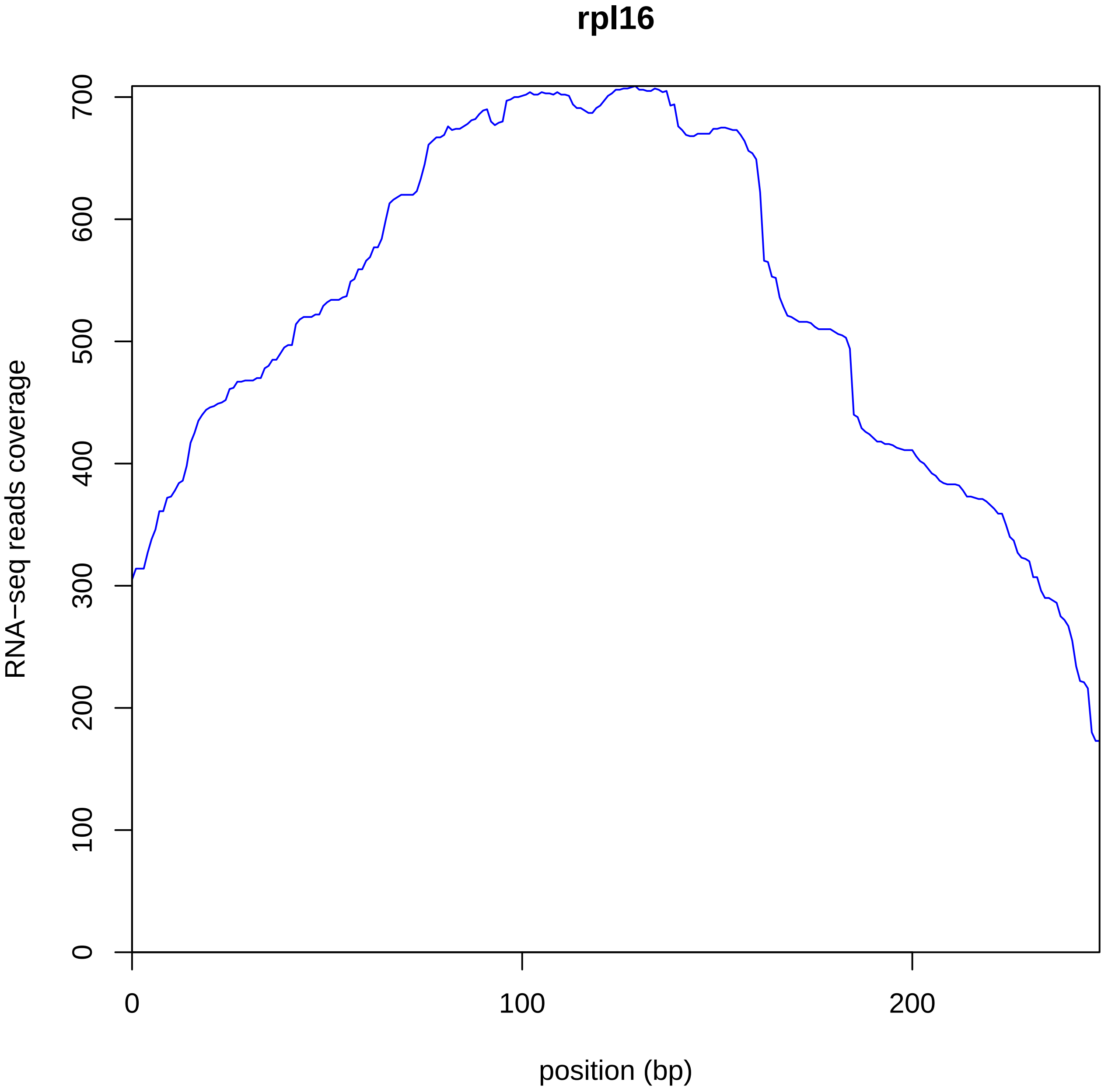

rpl16
RNA−seq reads coverage
0
100
200
300
400
500
600
700
0
100
200
position (bp)

AB

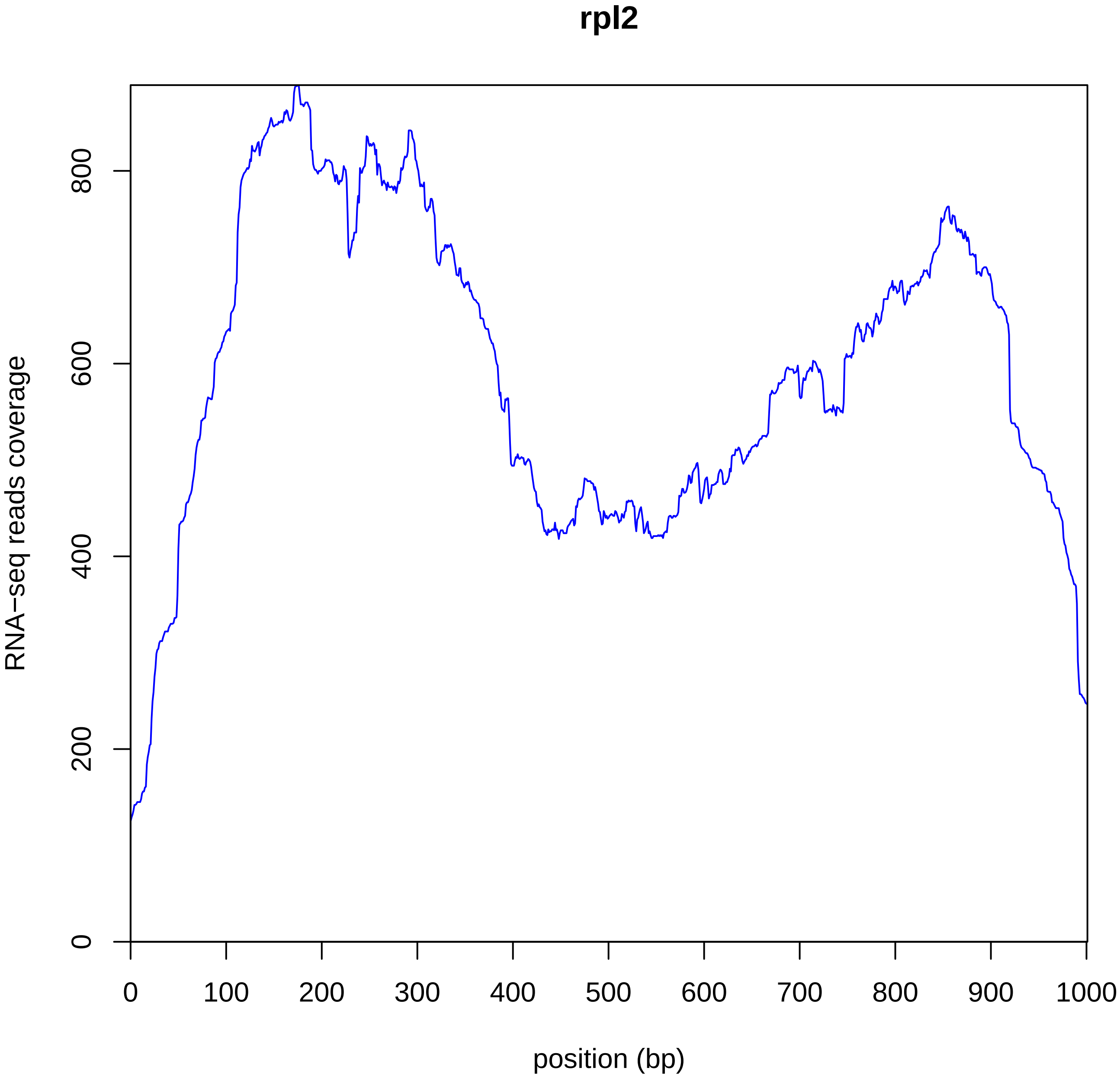
rpl2
RNA−seq reads coverage
0
200
400
600
800
0
100
200
300
400
500
600
700
800
900
1000
position (bp)

AC

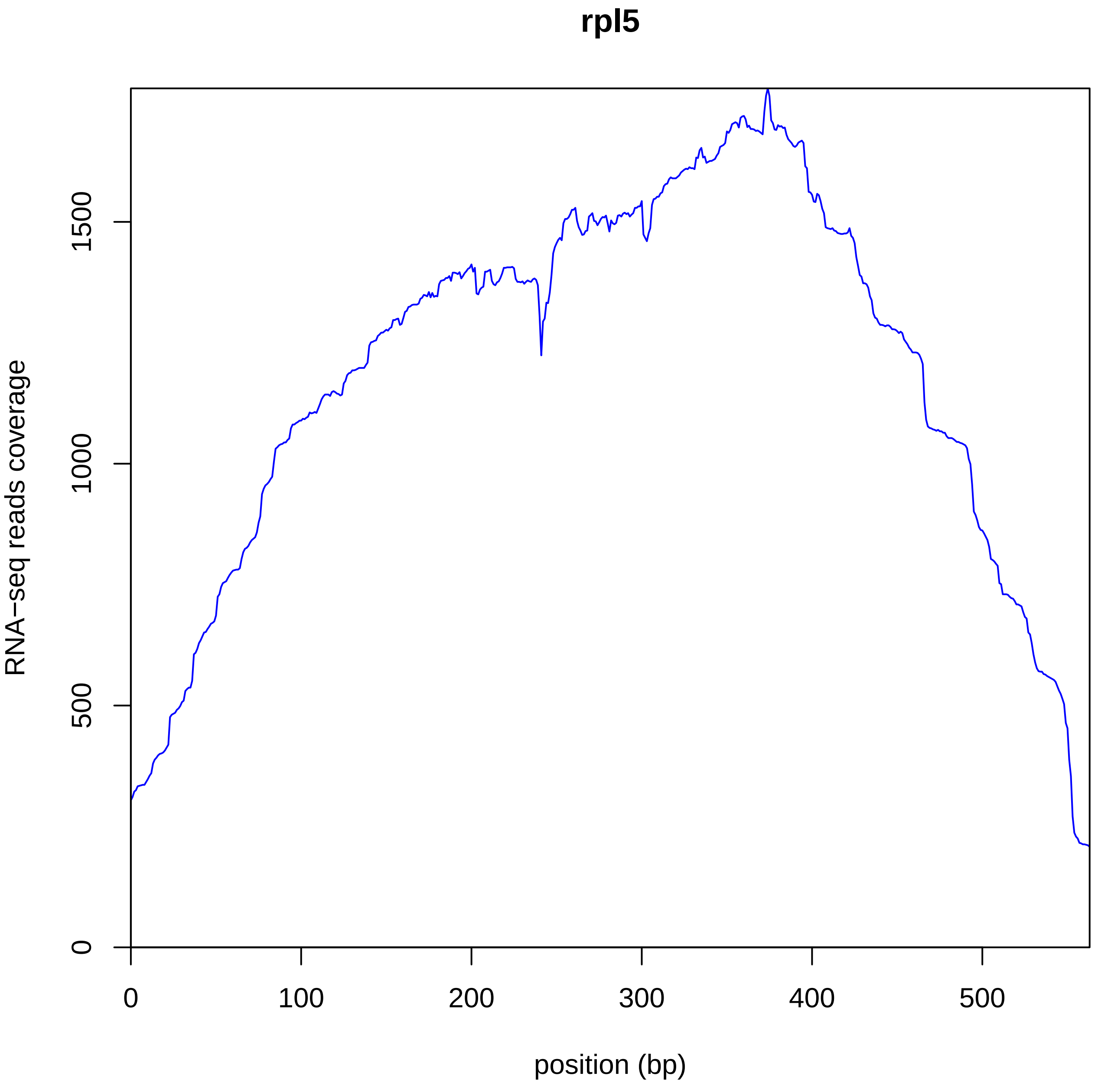
rpl5
RNA−seq reads coverage
0
500
1000
1500
0
100
200
300
400
500
position (bp)

AD

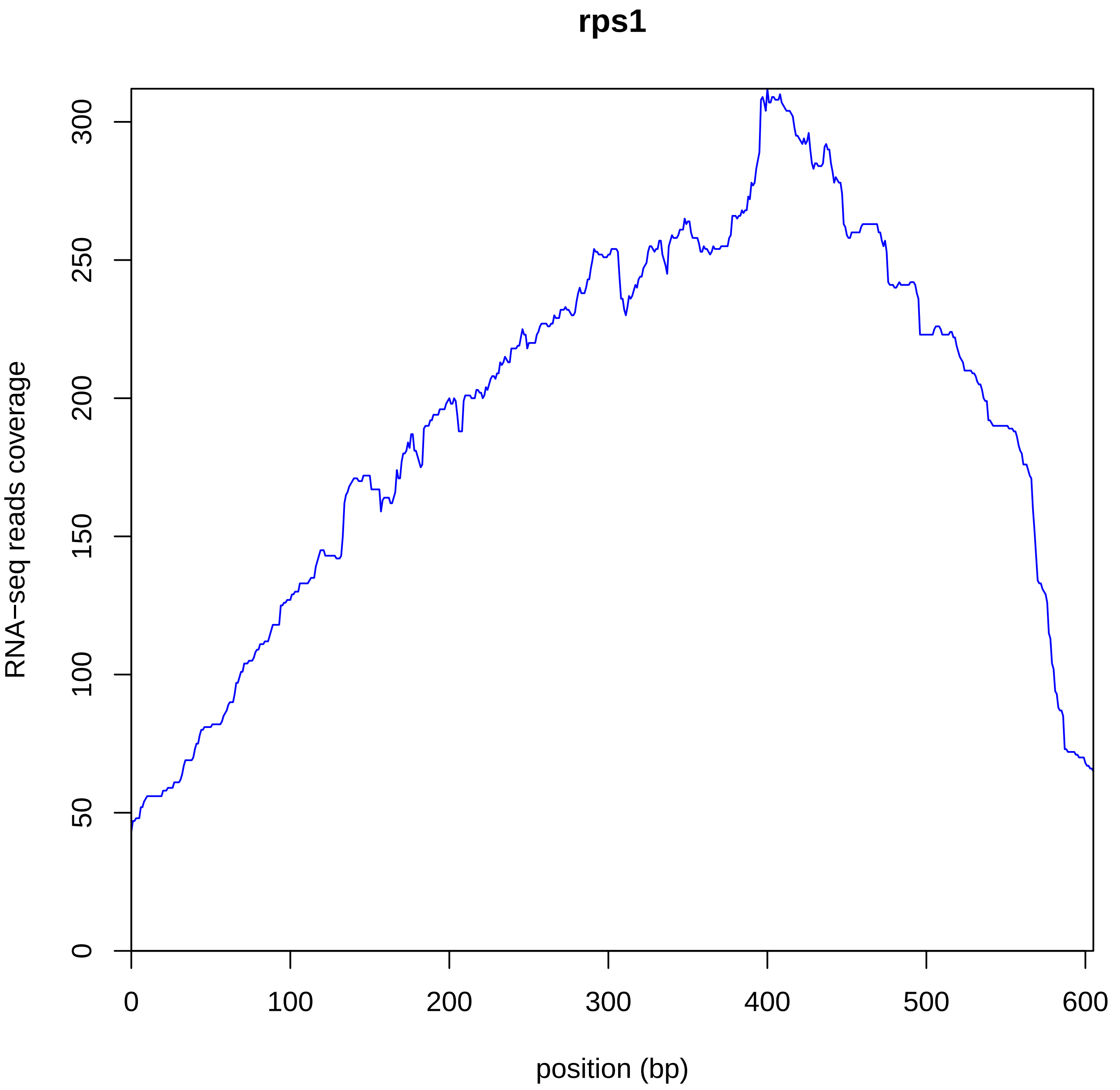
rps1
RNA−seq reads coverage
position (bp)
0
50
100
150
200
250
300
0
100
200
300
400
500
600

AE

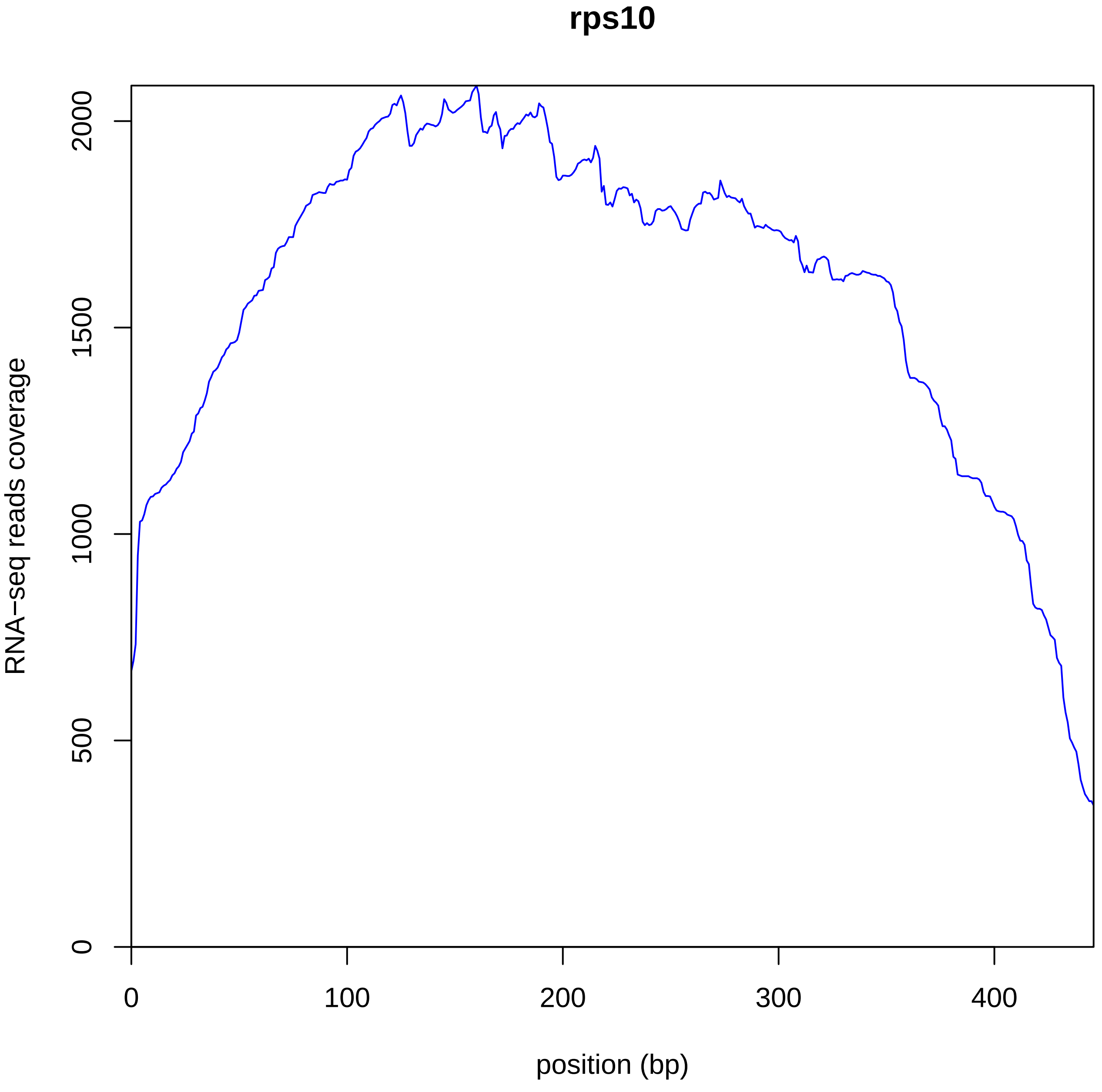

rps10
RNA−seq reads coverage
0
500
1000
1500
2000
0
100
200
300
400
position (bp)

AF

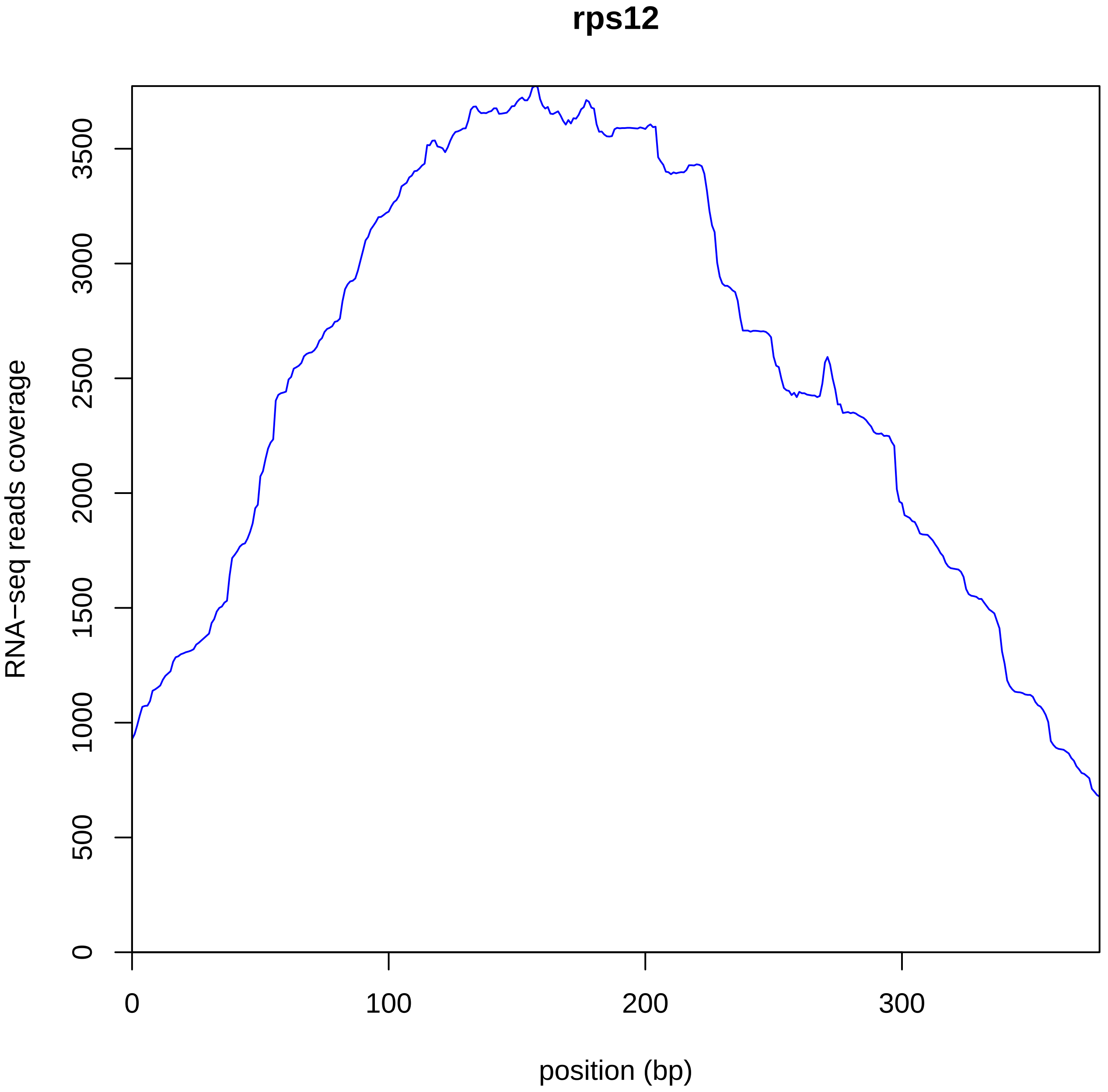
rps12
RNA−seq reads coverage
0
500
1000
1500
2000
2500
3000
3500
0
100
200
300
position (bp)

AG

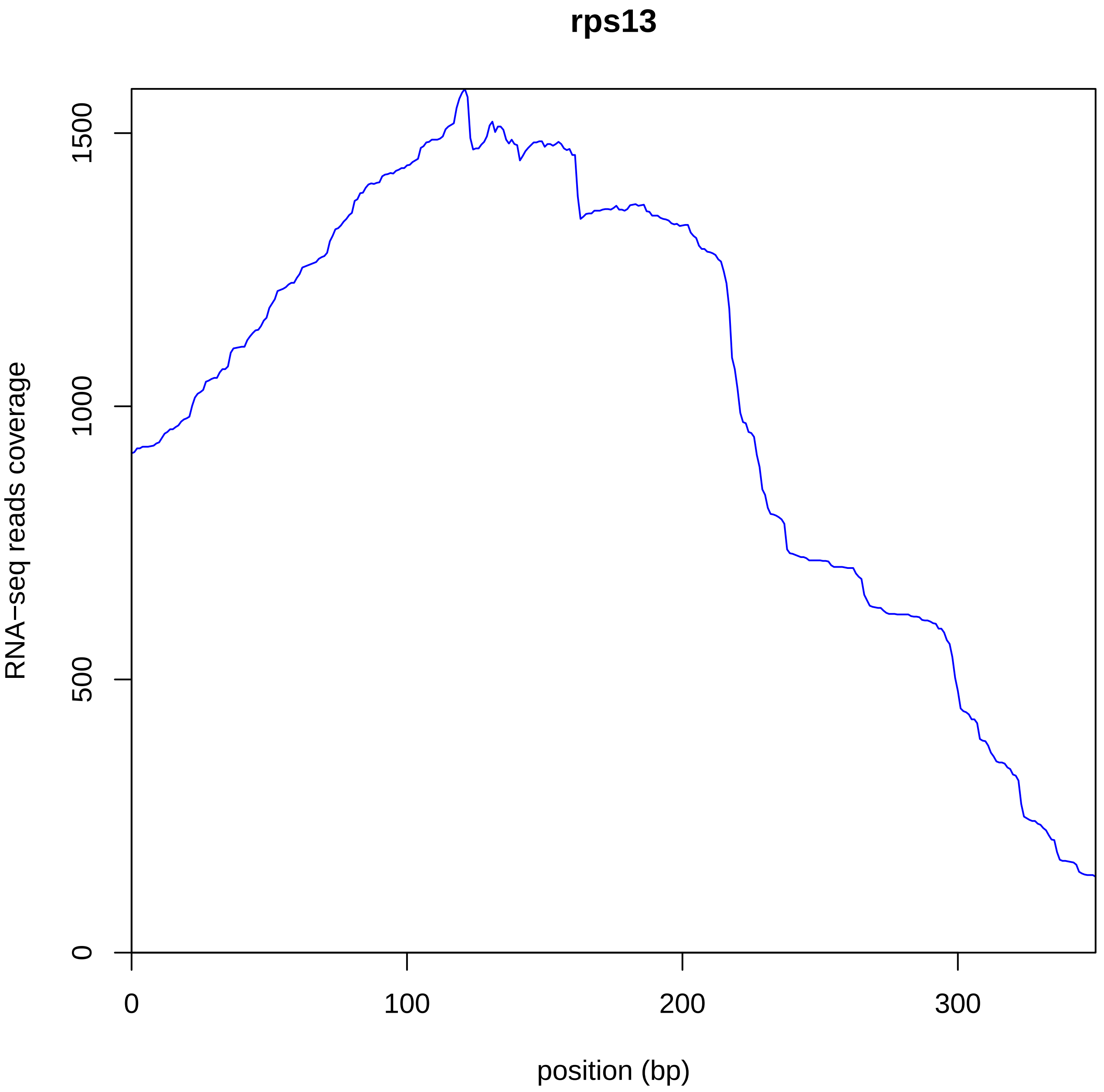

rps13
1500
1000
500
0
RNA−seq reads coverage
0
100
200
300
position (bp)

AH

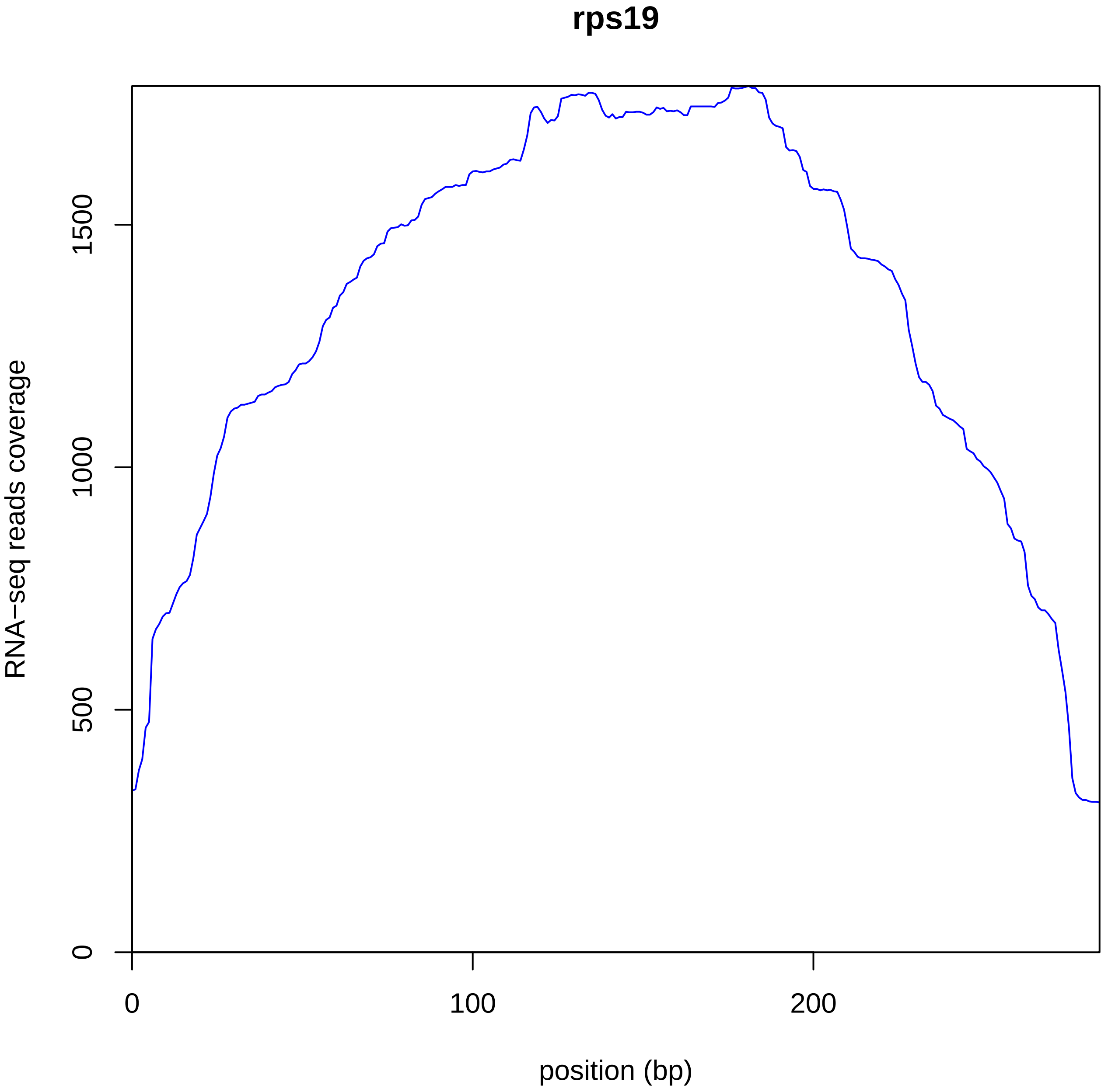
rps19
RNA−seq reads coverage
1500
1000
500
0
0
100
200
position (bp)

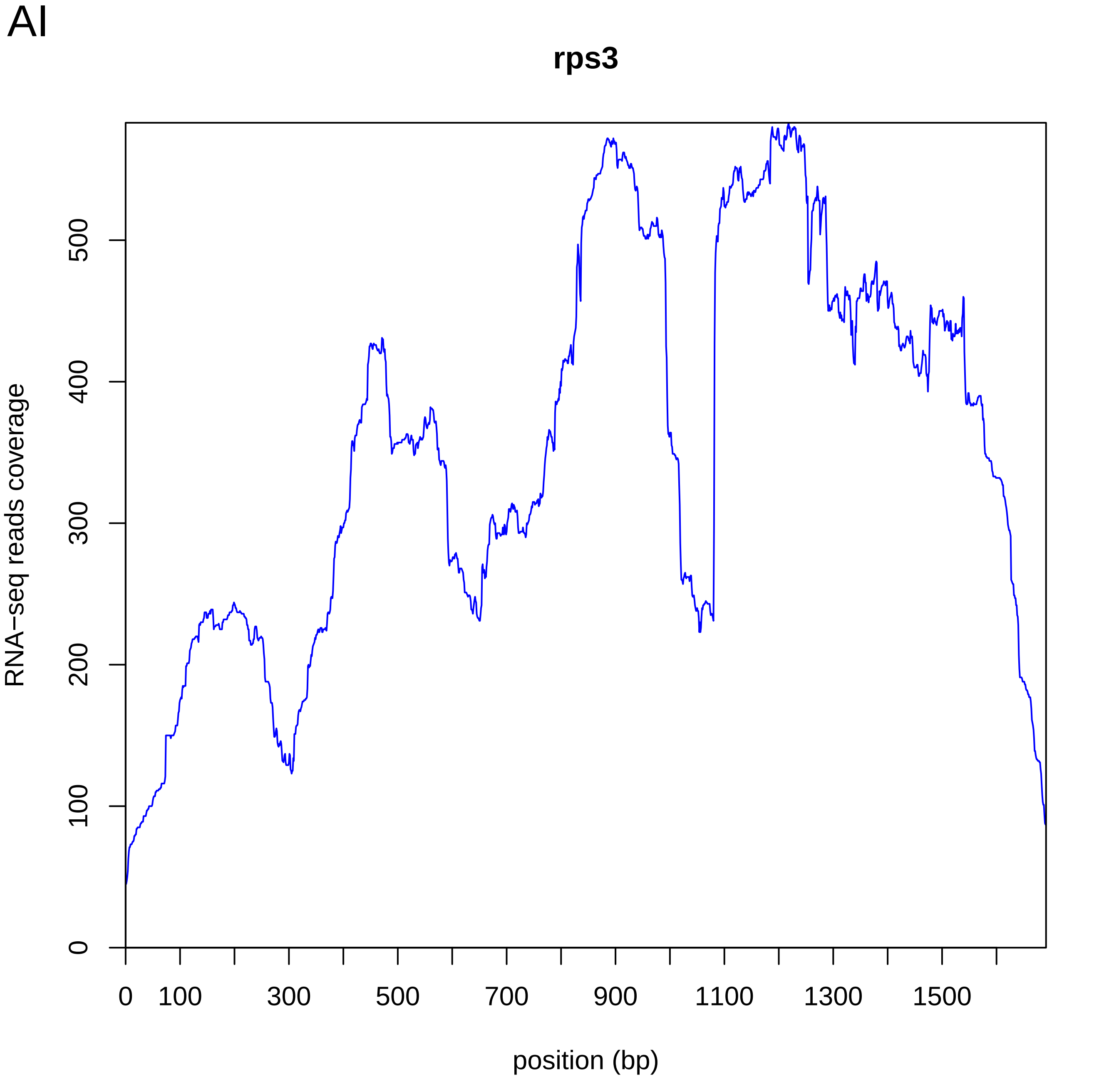
AI
rps3
RNA−seq reads coverage
0
100
200
300
400
500
0
100
300
500
700
900
1100
1300
1500
position (bp)

AJ

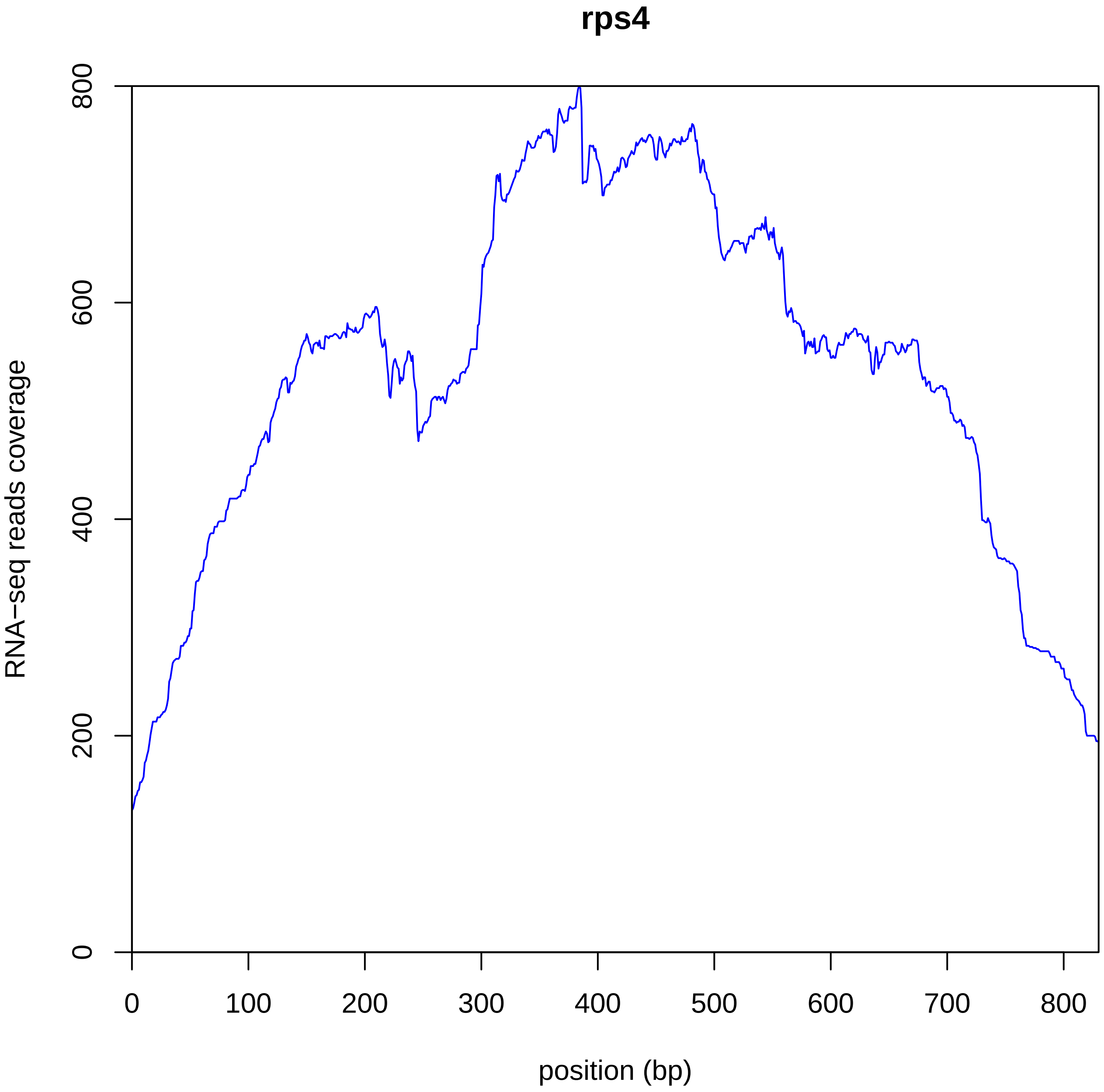
rps4
RNA−seq reads coverage
position (bp)
0
200
400
600
800
0
100
200
300
400
500
600
700
800

AK

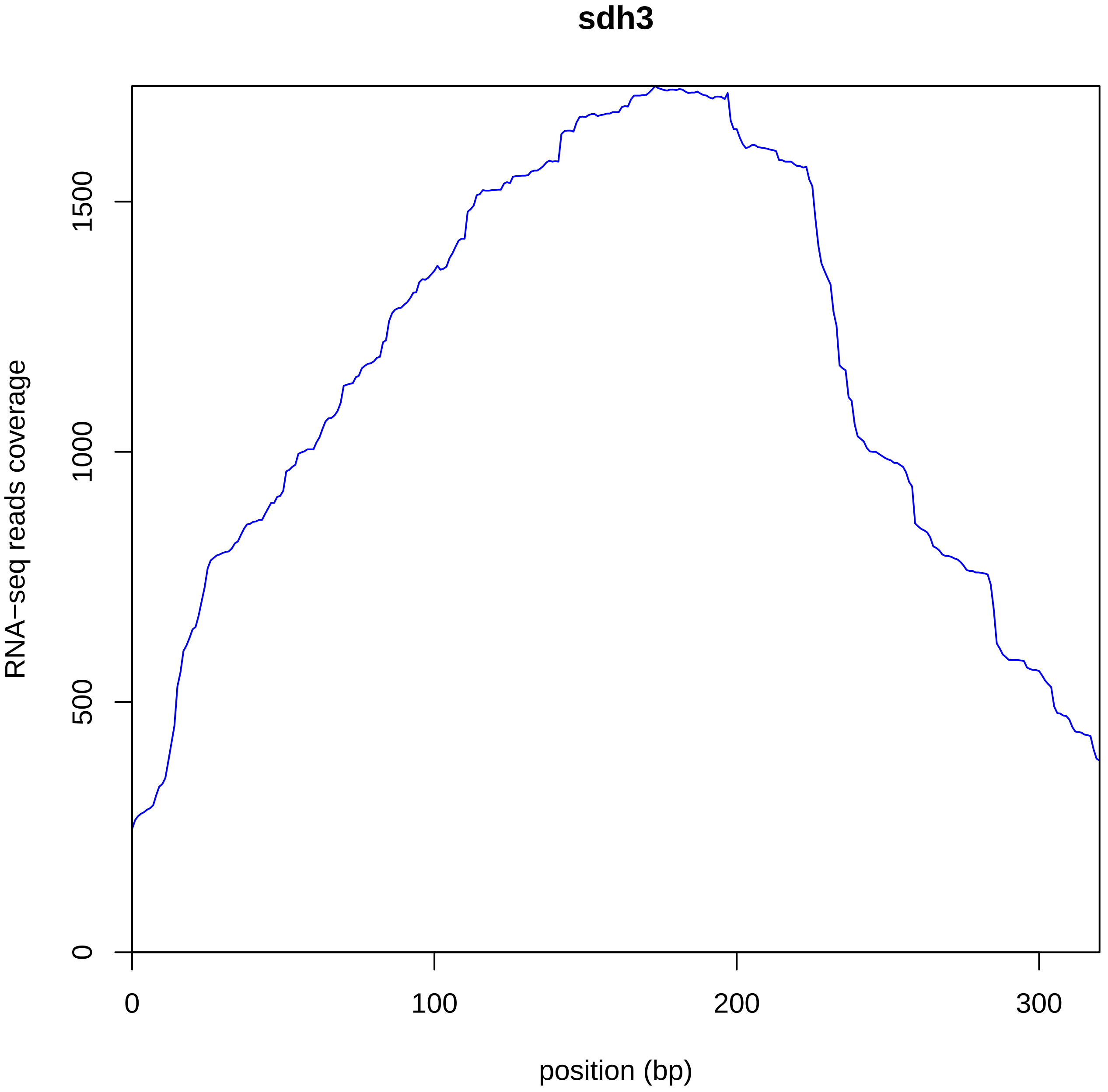
sdh3
RNA−seq reads coverage
0
500
1000
1500
0
100
200
300
position (bp)

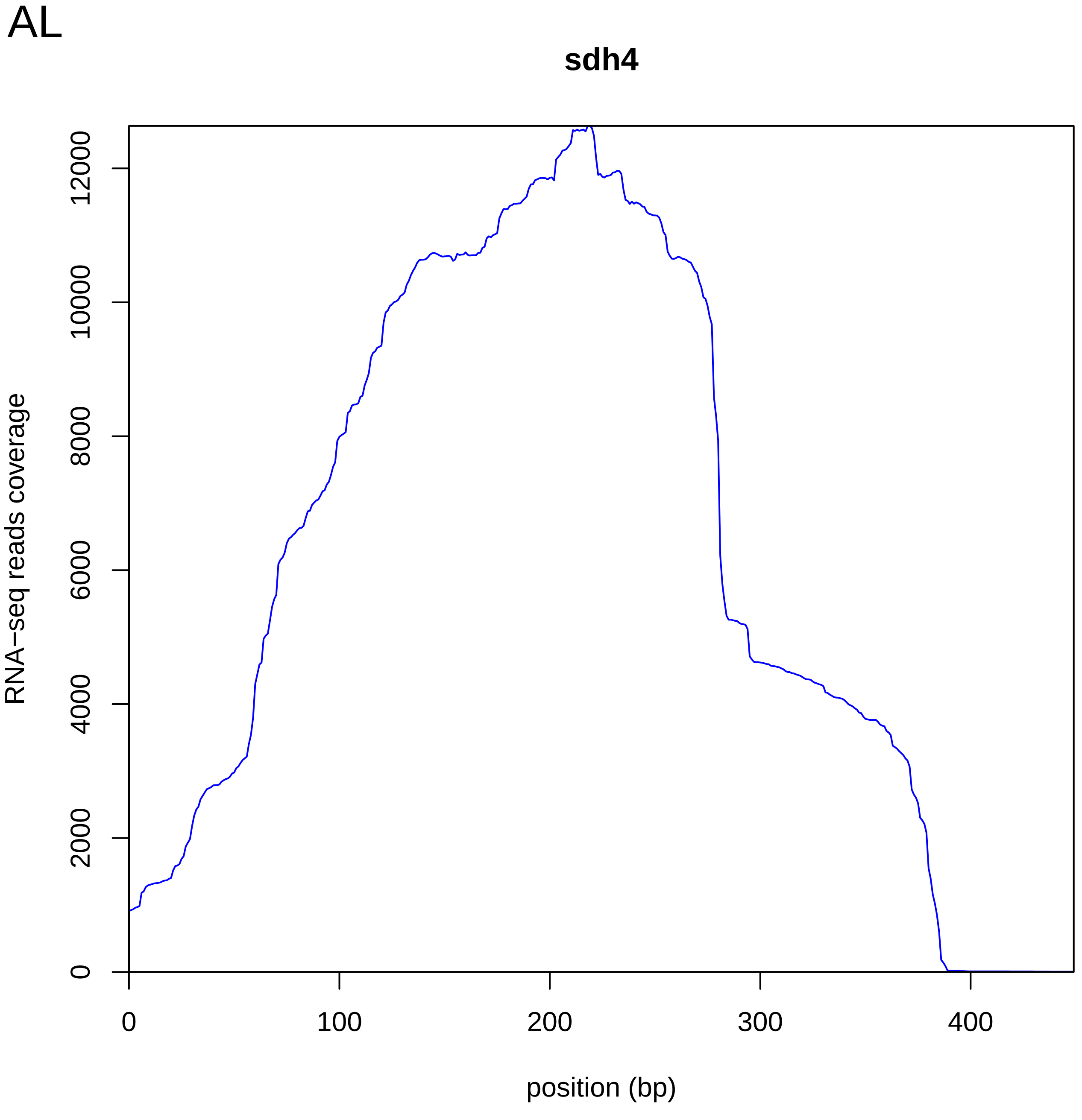
AL
sdh4
RNA−seq reads coverage
position (bp)
0
2000
4000
6000
8000
10000
12000
0
100
200
300
400
