# Supplementary figures and images for: Mitochondrial genome of the nonphotosynthetic mycoheterotrophic plant Hypopitys monotropa, its structure, gene expression and RNA editing
Source: PeerJ. 2020 Jun 19;8:e9309. doi: 10.7717/peerj.9309 (PMC7307570; doi:10.7717/peerj.9309)

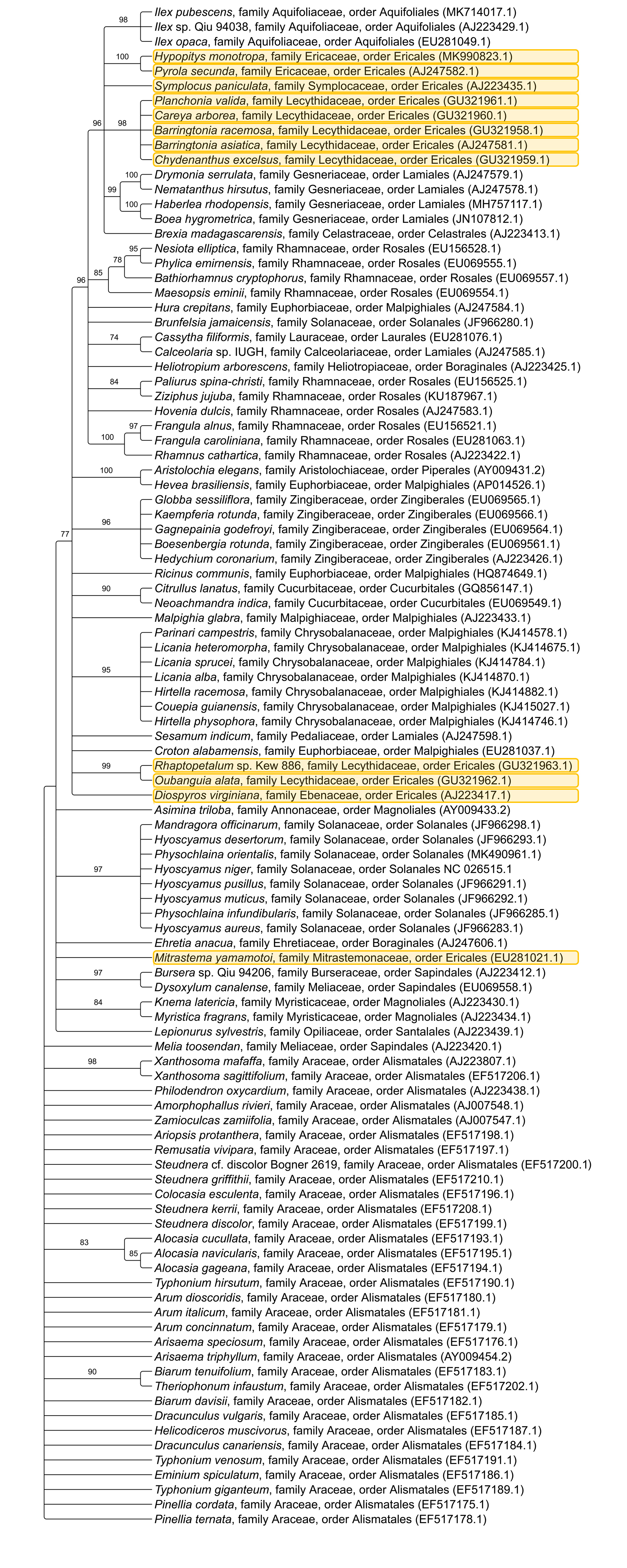

Supplement: Supplemental Information 10 [file peerj-08-9309-s010.jpg]
